# Monitoring Liquid Concentration Changes in Nanofluidic Channels Using Cross-Grating Wavefront Microscopy

**DOI:** 10.1021/acsnano.6c12559

**Published:** 2026-10-15

**Authors:** Lova Wilske, Baptiste Marthy, Puvaneswari Teluchina-Appadu, Joachim Fritzsche, Guillaume Baffou, Christoph Langhammer

**Affiliations:** † Department of Physics and Astronomy, 11248 Chalmers University of Technology, Gothenburg SE-412 96, Sweden; ‡ 131923 Institut Fresnel, CNRS, Aix-Marseille University, Centrale Med, Marseille 13013, France

**Keywords:** optical microscopy, nanofluidic systems, quantitative phase microscopy, cross-grating wavefront microscopy, refractive index, solute diffusion

## Abstract

Optical microscopy that employs intensity measurements of scattered, transmitted, or emitted light is critical to study micro- and nanofluidic systems widely used in, e.g., biology, chemistry, and materials science. Since such fluidic systems often are transparent, simple bright-field microscopy observations are bound to yield low-contrast images. Fluorescence labeling or Raman imaging can be used to overcome this limitation and yield high contrast. However, these approaches are invasive or complex and slow and do not yield direct quantitative information about a liquid inside a micro- or nanofluidic structure. Quantitative phase microscopy techniques on the other hand are very efficient in enhancing the contrast of transparent samples, in a noninvasive manner. However, they have never been applied to nanofluidic systems. To fill this gap, we demonstrate how cross-grating wavefront microscopy enables the quantitative characterization of liquids in terms of concentration and refractive index, as well as solute diffusion, inside a single nanofluidic channel embedded in a SiO_2_ matrix in general and from diffraction-limited nanofluidic channel volume fractions as small as ∼45 aL. In a wider perspective, this study thus demonstrates the applicability of cross-grating wavefront microscopy to the characterization of processes taking place in volumes smaller than the diffraction limit of light, such as, e.g., nanofluidic systems, nanopores, or nanocavities.

## Graphical abstract

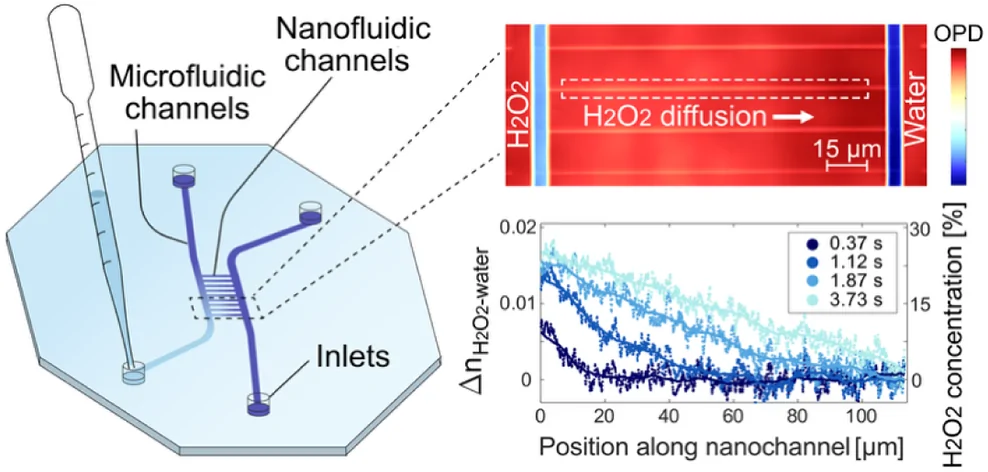

Microfluidic systems constitute an important platform for biologists, chemists, and materials scientists alike and have enabled breakthroughs in fields that include drug development,^1^ the understanding of cellular interactions,^2^ and the flow synthesis of liquid chemical products^3^ and nanocrystals.^4^ In parallel, spurred by the advances in nanofabrication methodologies during the past two decades, nanofluidics has emerged as a complement that enables studies down to nanometer length scales.^5–12^ This confinement in nanofluidic systems in general, and in nanofluidic channels in particular, provides a flexible platform for studying, trapping, and manipulating single nanoscopic entities, such as biomolecules and biological and inorganic nanoparticles. Furthermore, it unlocks precise control of fluid flow in the close environment of such nanoscale entities. Due to these prospects, over the years, numerous experimental methods have been developed and applied to characterize, e.g., fluid composition and flow inside and through nanofluidic structures, as well as nanoparticles and molecules inside them. These methods include mass spectrometry,^13^ chromatography,^14^ mass flowmetry,^15^ electrophoresis,^16–20^ resistivity measurements,^21,22^ and different microscopy^23–35^ and spectroscopy^36,37^ techniques.

On this list of methods, optical microscopy is particularly interesting due to its wide availability at reasonable cost, its versatility and relative ease of use, low invasiveness, *in situ* compatibility, and high spatial and temporal resolution. To this end, fluorescence microscopy^23,24,28–30^ in particular but also plasmonic sensing and nanoimaging,^13,38–40^ Raman microscopy,^25–27^ diffraction-based detection,^32–34^ and nanofluidic scattering microscopy (NSM)^31,35,41^ and spectroscopy (NSS)^37,42^ have been demonstrated to be useful for studying single molecules, nanoparticles and fluids, and their interactions, confined in nanofluidic structures. To this end, it is important to highlight a key advantage of nanofluidic systems in this respect, i.e., their ability to confine analyte fluids, molecules, and particles essentially in two dimensions and thus retain them within the focal plane of an optical microscope for extended periods of time, while not requiring surface attachment of the studied nanoentities. This is critical as it on one hand lowers the limit of detection and/or the characterization accuracy and on the other hand ensures that, for example, biological species are in their natural state. As a second aspect, we note that the microscopy techniques summarized above rely either on the emission of visible light, e.g., from a fluorophore, or on light either scattered from the nanoscopic objects inside the fluidic structure, from the nanofluidic structure itself, or from interference effects between the two. In other words, they rely on measured intensity changes of light emitted by the sample. However, there is a second dimension to using light as a probe—its phase—as done in quantitative phase imaging (QPI) techniques, which traditionally have been used in, e.g., the study of living cells or liquid droplets,^43,44^ and also have found application in microfluidics.^45–49^ However, as a key point here, they have not yet been applied to nanofluidics. This distinction is nevertheless important because in the microfluidic regime, the fluidic structures are larger than the diffraction limit of visible light, while they typically are smaller than that limit in the nanofluidic regime.

At the conceptual level, QPI relies on light being transmitted through a medium experiencing a phase shift, whose magnitude is proportional to the difference in refractive index (RI) between the medium transmitting the light and its surroundings. Among the QPI methods, cross-grating wavefront microscopy (CGM), which is based on quadriwave lateral shearing interferometry (QLSI),^50^ stands out due to its compactness and ease of integration with a wide range of optical microscopes.^51^ At its core, it employs a two-dimensional checkerboard-structured cross-grating that is placed in front of a camera sensor to record interferograms that encode wavefront distortion through the intensity of light diffracted by the grating.^52^ Owing to its high sensitivity and low noise level, CGM has pioneered applications of QPI in nanophotonics. For example, it has been used to study photothermal effects in plasmonics,^53^ to characterize metasurfaces,^54^ and to determine the complex polarizability of nanoparticles^55^ and 2D materials.^56^ However, QPI has never been used in combination with nanofluidic structures. Here, we take this step and employ CGM to investigate individual nanofluidic channels and the liquid exchange and solute diffusion inside them, from liquid volumes that can correspond to diffraction-limited fractions of a single nanochannel with geometrical volumes as small as ∼45 aL for the specific investigated nanochannel cross-sectional area of ∼250 × 350 nm^2^. We show that in such channels with geometrical cross-sections smaller than the diffraction limit, a high phase contrast is obtained that is proportional to the RI of the liquid in the nanochannel and that this proportionality can be used to determine absolute solute concentrations in the 250 mM range, as well as to visualize flow and diffusion profiles, as exemplified by optically transparent nonabsorbing aqueous NaCl and H_2_O_2_ solutions.

## Results

We designed a fluidic chip that comprises a microfluidic in- and outlet system with four connection points for macroscopic liquid handling via a custom chip holder that interfaces the chip to a Fluigent MFCS-EX pressure regulator used to control the liquid flow. The microfluidic system is connected to an array of parallel straight nanofluidic channels fabricated into a 2150 nm-thick thermally grown oxide layer on a Si wafer. It is composed of 16 parallel channels in four repeated sets of 250, 175, 140, and 105 nm depth, respectively, with a constant width of 350 nm (Figure 1a–d; see Methods for details).

**Figure 1:**
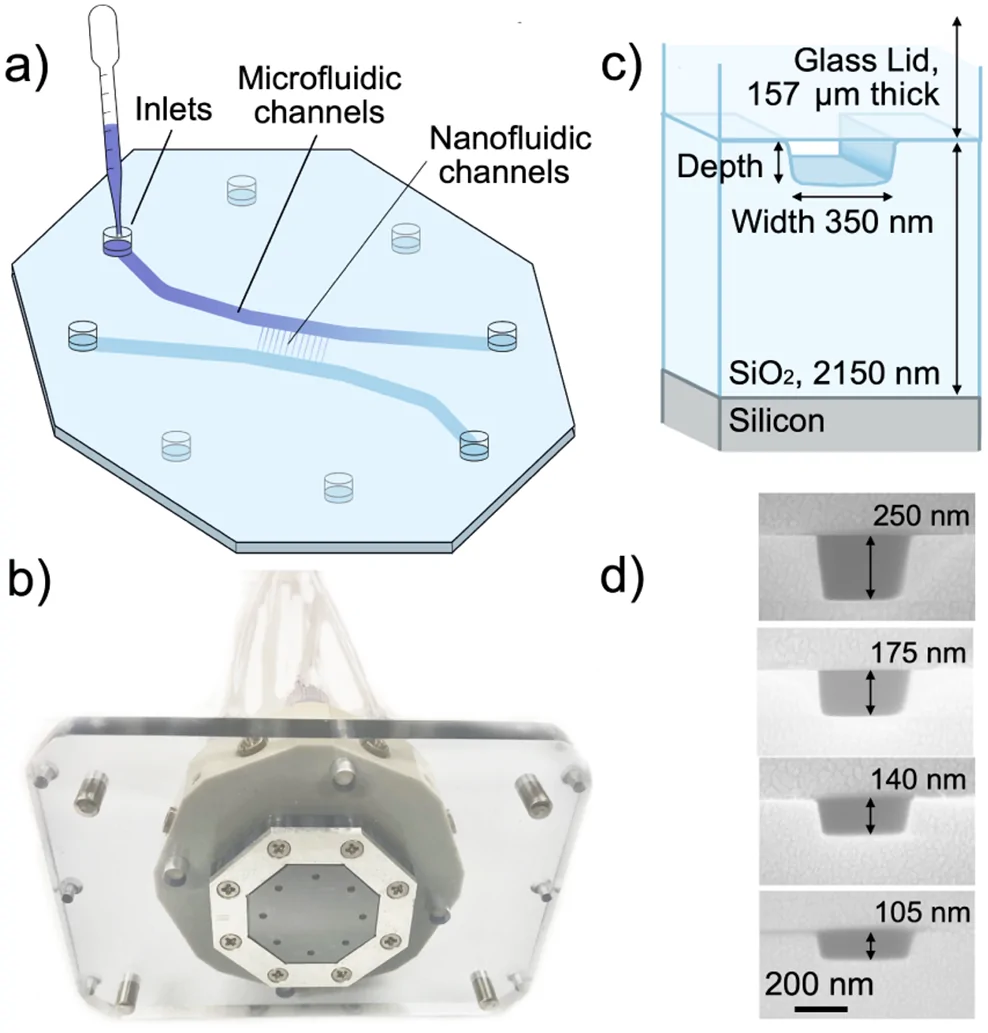
a) Schematic of the fluidic chip. Nanofluidic channels are fabricated in an array in the middle of the chip and are connected to a microfluidic channel on each end. The microfluidic channels are in turn accessed via inlet holes. Only 4 of the fluidic chip’s total 8 inlet holes are used in this study. b) Photograph of a fluidic chip mounted in its holder. Gas tubes that can be pressurized are connected to the backside of the holder to enable convective flow in a desired direction by applying pressure on the corresponding inlets. c) Schematic illustration of the cross-section of a nanochannel etched into the 2150 nm-thick thermal oxide layer (SiO_2_) grown on the Si wafer substrate with characteristic dimensions indicated. A glass lid is thermally bonded to the SiO_2_ layer to seal the fluidic system. d) SEM images of representative nanochannel cross-sections of the four different depths used in this work.

In CGM, a cross-grating is placed at a millimeter distance from the sensor of a camera to enable measurements of phase shifts of visible light when it travels through the sample plane of a microscope.^51,52,57^ To extract the phase shift, we record two interferograms—one with the object of interest and a second one of an empty region of the sample, i.e., a uniform region without fluidic channels, which serves as a reference (Figure 2). Specifically, the phase shift can be retrieved from the interferograms using an algorithm based on Fourier transforms.^52,57,58^ Here, we used a setup with a narrow-band noncoherent red LED light source (center wavelength 620 nm), in a Köhler illumination configuration. The sample was imaged in reflection mode (Figure 2a—see Methods for details), with a 40× oil immersion objective, such that light propagates through the borosilicate glass lid of the fluidic chip, through the thermal SiO_2_ layer down to the Si wafer substrate where it is reflected back and travels through the sample a second time. The collected light is directed through a tube lens before it reaches the cross-grating, where it is diffracted. This diffracted light then forms an interference pattern (interferogram) that is recorded with a CMOS camera. Since the camera housing prevents direct mounting of the cross-grating onto the camera sensor, we use a relay lens (4f configuration) to reimage the grating in front of the camera (see Figure 2a).

**Figure 2:**
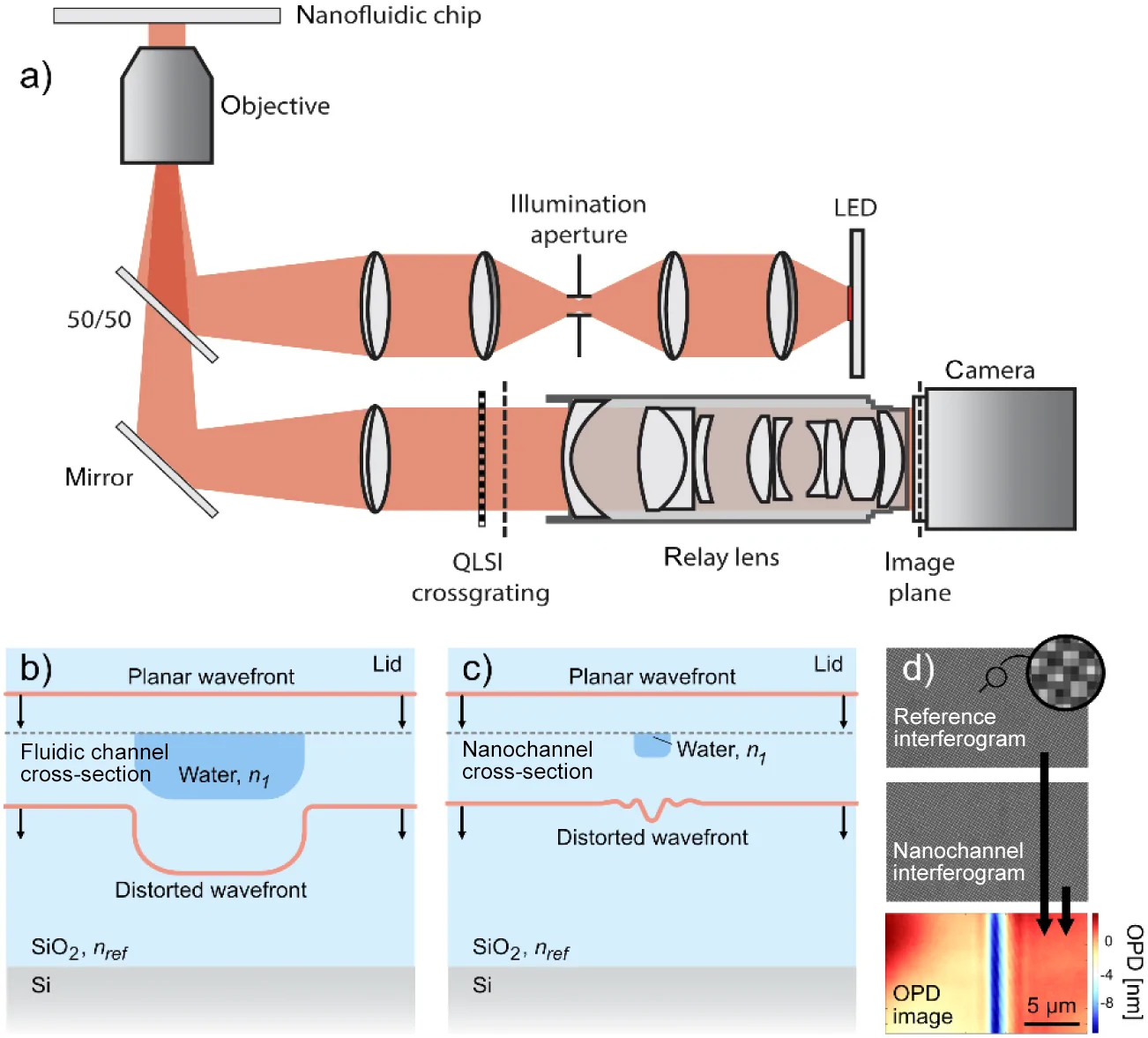
a) Schematic depiction of the CGM configuration used for reflection-mode measurements of nanofluidic systems. b) Sketch of a planar wavefront that is distorted as it is transmitted through a microfluidic channel with cross-sectional dimensions larger than the diffraction limit of light. c) Sketch of the same scenario but for a nanofluidic channel that is smaller than the diffraction limit of light. Here, the wavefront distortion caused by the nanochannel is smeared out by the point spread function of the microscope system. d) In CGM, OPD images are formed by comparing the interferograms of a sample frame and a reference frame using a tailored algorithm. These interferograms are processed to provide the optical path difference (OPD) in nm. The displayed OPD example image corresponds to a nanochannel with a 175 × 350 nm^2^ cross-sectional area.

When a planar wavefront is transmitted through such a fluidic chip, the area occupied by fluidic channels will induce a distortion to the wavefront, while the remaining empty area will leave it unaltered. If we first consider a scenario where the fluidic channel is much *larger* in diameter than the diffraction limit of light (Figure 2b), the distortion of an incoming planar wavefront transmitted through the fluidic channel at a given point in space (*x,y*) can be quantified as an optical path difference (OPD), δ*l*. This OPD is proportional to the distance the light has traveled through the channel, which in a reflection-mode measurement as in the present case corresponds to twice the channel depth, and the RI difference between the RI of the medium inside the channel, *n*(*x,y*), and the RI of the medium embedding the channel, *n*_ref_, according to

1 $$\delta l\left(x,y\right)=\left(n\left(x,y\right)-n_{ref}\right)dz$$

Hence, a change in the RI of the medium inside the channel, Δ*n*, will induce a change in the OPD that can be measured and used to provide information about a process of interest that occurs inside the channel.

From the OPD, one can calculate the optical volume (OV), δ*V* as the integral of the OPD image:

2 $$\delta V=\iint_{S}\delta l\left(x,y\right)dxdy$$

In the case *n* is uniform, this equation becomes

3 $$\delta V=\left(n-n_{\mathrm{ref}}\right)V$$

where *V* is the geometrical volume of the object. Computing the OV is common in optical QPI to extract, e.g., the dry mass of living cells^57^ or the optical properties of nanoparticles.^55^ Note that this description, and especially eq 1, is only valid when ray optics apply, i.e., when the dimensions of the imaged object are much larger than the wavelength of light. In the context here, this is true for a microfluidic channel but, as the key point, it is not true for a nanofluidic channel. Hence, for the case of an object smaller than the diffraction limit of light (the lateral resolution of the microscope is r = λ/(NA_obj_ + NA_ill_) ≈ λ/NA_obj_ = 480 nm), such as a nanofluidic channel, an induced phase shift will be smeared out by the point spread function (PSF) of the system (Figure 2c). However, by integrating the OPD in eq 2 over a sufficiently large area that encapsulates the diffraction rings, this expression still returns a proper estimation of the OV that can be used, for instance, in eq 3 to calculate the RI, *n*, as demonstrated for nanospheres.^55^ The corresponding mathematical expression reads as

4 $$n=\frac{\delta V}{V}+n_{ref}$$

and enables the measurement of the RI of a liquid contained in a nanofluidic channel with subwavelength cross-sectional dimensions. For this work, since we operate the microscope in reflection mode, a factor of 2 is required in eq 4 since light crosses the imaged object twice. The geometrical volume, *V*, of a nanochannel is V = 2*AL*, where *L* *is* the channel length covered by the integration surfaces *S* in eq 2 and *A* is the cross-sectional area of the channel, which can be determined by SEM image analysis.

To demonstrate the ability of CGM to measure RI changes inside nanofluidic channels, we performed measurements on two liquids with well-defined RI: water and a 3 M aqueous NaCl solution. We used a pressure regulator to fill a nanochannel with the respective liquids via convective flow and recorded the corresponding OPD images (Figure 3a,b). In the OPD images, the nanochannels appear as bright vertical lines, and the microchannels, which the nanochannels are connected to, cover a larger area in the horizontal direction of the image. The two OPD images in Figure 3a and b are obtained with the same reference interferogram, which is acquired from an area on the chip without fluidic structures. A single nanochannel with 175 nm depth when filled with water reveals an OPD peak value of around −12 nm (Figure 3c; see Figure 4a for identical images for all four nanochannel depths), and the same nanochannel filled with a 3 M NaCl solution exhibits an OPD peak value of around −9 nm (Figure 3d) (see Methods for a description of the applied background subtraction). This difference becomes even clearer when visualizing the change in OPD induced by introducing the 3 M NaCl solution to replace the water by plotting OPD profiles *across* the nanochannel (Figure 3e). We also note that, as a consequence of diffraction, the displayed OPD profile does not reach the OPD level expected from ray optics (which would be 45.5 nm for water and 35.8 nm for the 3 M NaCl solution in a 175 nm-deep channel), described in eq 1.

**Figure 3:**
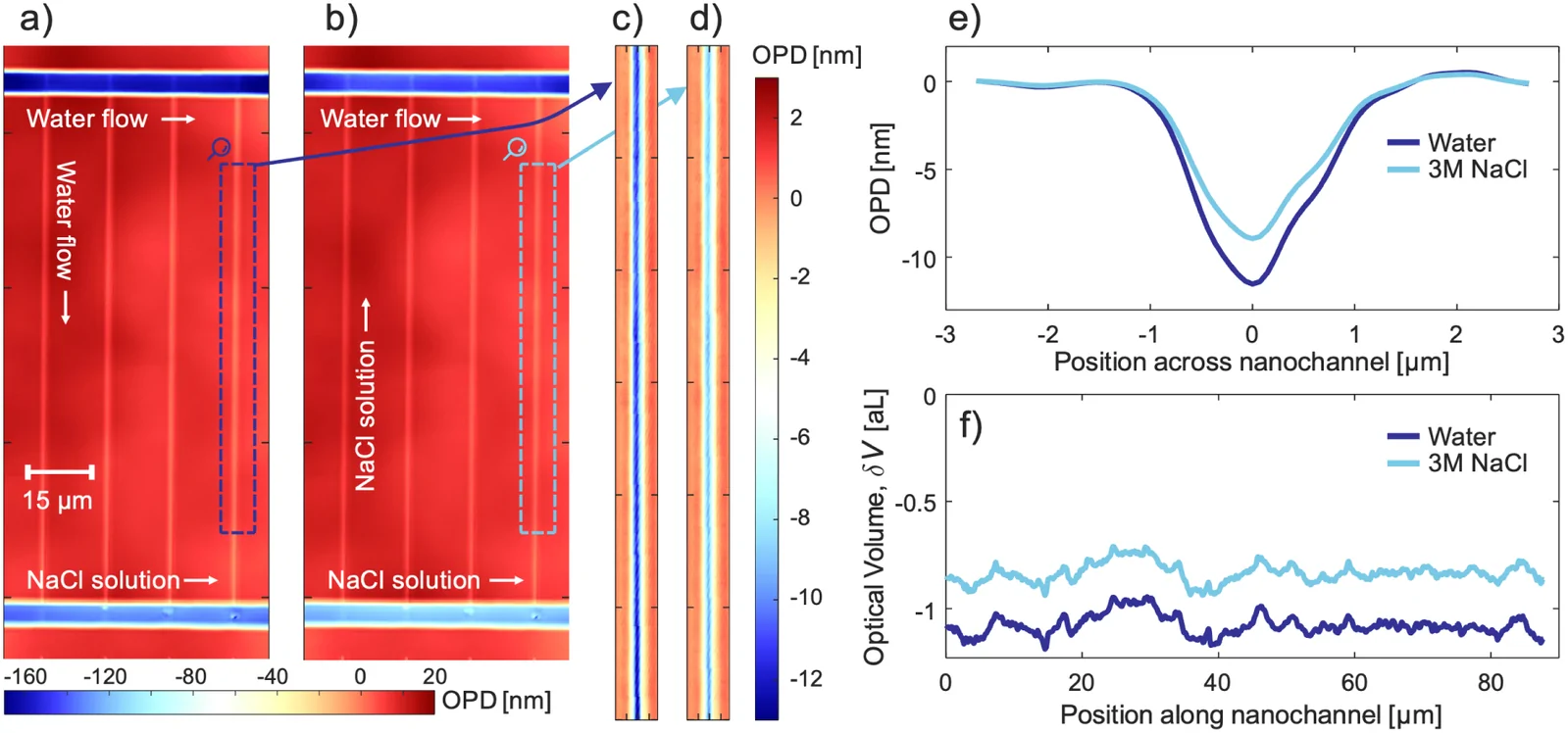
OPD images of vertically aligned nanofluidic channels with nominal 350 × 175 nm^2^ cross-sections, which are below the limit of diffraction of light, filled with water (a and c) and a solution of 3 M NaCl (b and d). The nanochannels are connected to horizontal microfluidic channels with a 5 × 0.97 μm^2^ cross-section, which can be seen in a) and b). e) The average OPD cross-cut of the nanochannel displayed in c) and d). Since the RI difference between water and SiO_2_ the nanochannel is embedded in is larger than the RI difference between a 3 M NaCl solution and SiO_2_, a water-filled nanochannel yields a larger OPD signal than the corresponding case of a channel filled with a 3 M NaCl solution. f) δ*V* calculated for each pixel along the entire nanochannel presented in c) and d) for the channel being filled with water and with the 3 M NaCl solution, respectively.

**Figure 4:**
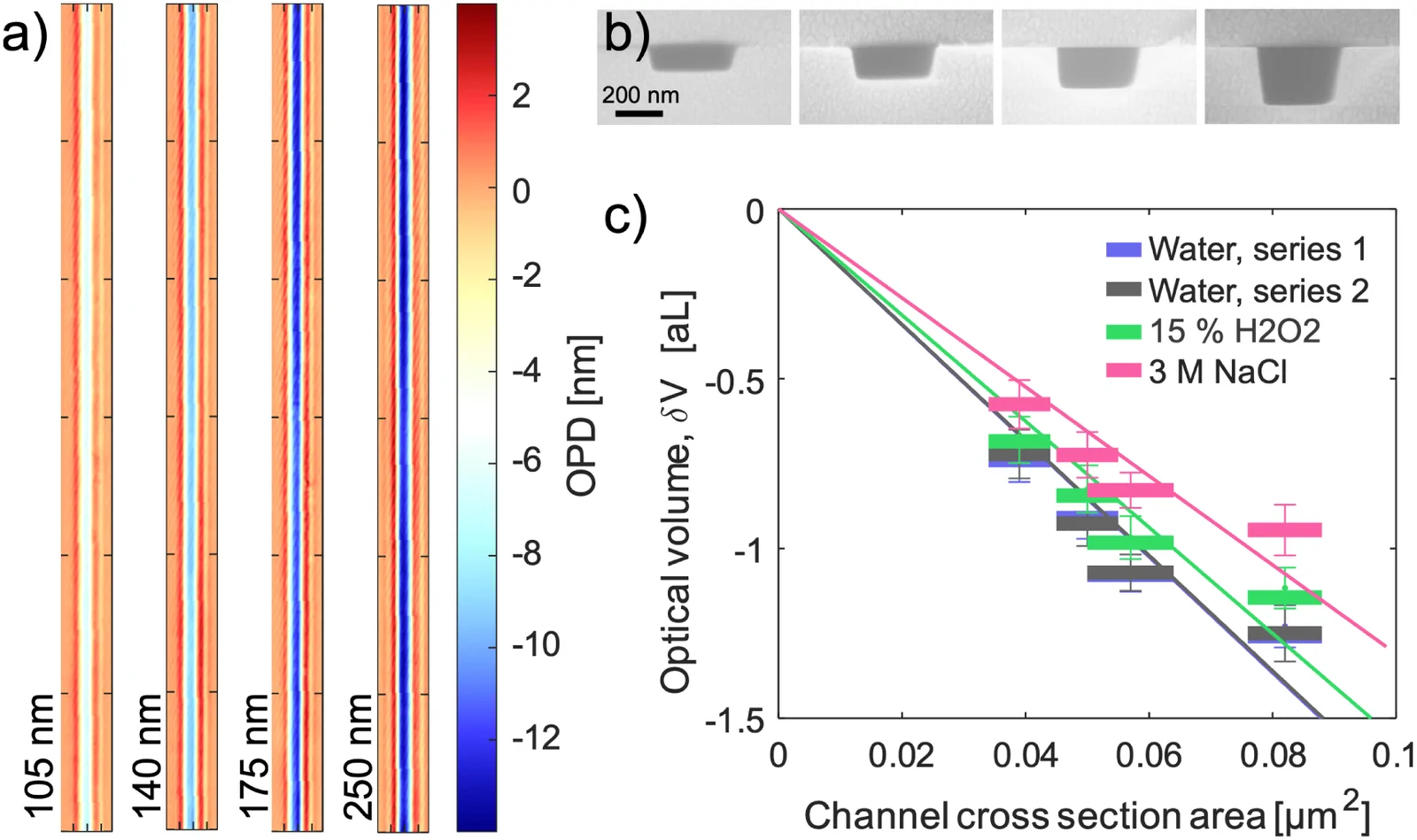
a) OPD images of water-filled 87 μm-long sections of single nanochannels of a constant350 nm nominal width and 105, 140, 175, and 250 nm nominal depths, respectively. Evidently, the OPD increases for larger channel depth. b) Representative cross-sectional SEM images of nanochannels on the same chip with nominally identical dimensions as for the channels used for the experiment depicted in a). As discussed in the text and SI section S1, the level of accuracy in cross-section area determination is limited due to SEM spatial resolution and charging effects during the imaging. c) Average OV along a 87 μm-long channel section for nanochannels filled with water, a 15% aqueous H_2_O_2_ solution, and a 3 M aqueous NaCl solution, respectively, with corresponding RIs *n*_water_ = 1.332, $n_{15\%H_{2}O_{2}}$ = 1.342, and *n*_3M,NaCl_ = 1.358. Each data point is based on measurements from 4 nominally identical nanochannels. The vertical error bars depict the OV standard deviation obtained from these 4 imaged channels. The length of each marker along the *x*-axis depicts the maximal and minimal nanochannel cross-section area obtained from image analysis of several cross-sectional SEM images of nominally identical nanochannels on the same chip (see SI section S1). For the measurements in water, two independently obtained data sets are included and depicted with blue and black markers. The solid lines depict linear regression forced through the origin.

As a next step, we calculate the OV *along* the entire 87 μm-long imaged nanochannel section (Figure 3f; see also SI Figure S5 for all four nanochannels with different depths) using eq 2, where each pixel corresponds to a 72.7 × 72.7 nm^2^ area of the fluidic chip. Comparing the OV obtained for the channel filled with water and the 3 M NaCl solution, respectively, we note a correlation in the OV and pixel position along the channel. The correlated unevenness observed in both OV measurements likely stems from structural defects within the nanochannel, like local surface roughness or small debris stuck in the channel that mediate the absolute OV along the channel in the same way both in the water- and NaCl-solution-filled state. However, if the *difference* in OV between the 3 M NaCl- and water-filled state, Δδ*V* = δ*V*_3__M,NaCl_ – δ*V*_water_, is extracted, the influence of said defects is eliminated. For the OV measurements presented in Figure 3f, this difference was on average Δδ*V* = 0.244 ± 0.016 aL for each pixel along the entire channel.

To more extensively investigate the ability of CGM to characterize nanofluidic systems, we applied the same procedure as described above to measure the OV of nanochannels with varying cross-sectional dimensions. To do so, we first generated OPD images in the water-filled state (*n*_*water*_ *=* 1.332) of the set of four parallel nanochannels with a constant width of nominally 350 nm and nominal depths of 250, 175, 140, and 105 nm, respectively (Figure 4a,b). This reveals a distinct decrease in the absolute OPD for the shallower channels, as expected. Repeating this procedure for the four channels with two additional and optically transparent solutions, i.e., a 15% aqueous H_2_O_2_ solution $\left(n_{15\%,H_{2}O_{2}}=1.342\right)$ and a 3 M NaCl aqueous solution (*n*_3_ *_M,NaCl_* = 1.358), and extracting the corresponding OV values and plotting them as a function of nanochannel depth reveal a reasonable linear dependence for all three solutions (Figure 4c). Furthermore, the absolute OV values for each nanochannel depth are the largest for the solution with the smallest *n*, i.e., water, as it exhibits the largest RI difference compared to the SiO_2_ (*n*_*SiO*2_ = 1.46 ^59^) that embeds the nanochannels. The vertical error bars depict the OV standard deviation obtained from identical measurements in 4 nominally identical nanochannels for each depth. Finally, we note that the level of accuracy in nanochannel cross-sectional area determination is limited due to SEM spatial resolution and charging effects during the imaging. To indicate this in Figure 4c, the length of each marker along the *x*-axis depicts the maximal and minimal nanochannel cross-section area obtained from image analysis of several cross-sectional SEM images of nominally identical nanochannels on the same chip (see SI Figures S1–S4 in section S1).

To further analyze these results with the aims to (i) extract *absolute RI difference* values induced by exchanging a liquid and to (ii) understand the accuracy and limitations of this method, we calculate the change in OV, i.e., Δδ*V* = δ*V_solution_* – δ*V*_*water*_, for nanochannels as we exchange the liquid inside them. We prepared aqueous solutions with RI close to the RI of water to explore the limit of detection, i.e., aqueous H_2_O_2_ solutions with concentrations ranging from 7.5% to 30% and aqueous NaCl solutions with concentrations ranging from 0.1 to 1 M. The corresponding measurements of Δδ*V* averaged over the imaged 87 μm-long nanochannel section are presented in Figure 5a (H_2_O_2_) and Figure 5b (NaCl). They yield a distinct linear dependence on concentration. The two OPD images used for the δ*V*_water_ and δ*V*_solution_ calculation for each set of channels were obtained using the same reference interferogram. Intermediate steps of the OPD image treatment, such as OPD cross-cuts of each channel and Δδ*V* values extracted along each channel, are depicted in SI Figures S6–S9. We also note that the error bars in Figure 5a,b depict the standard deviation of Δδ*V* *along* the entire imaged 87 μm-long nanochannel section for each channel depth and highlight that Δδ*V* varies slightly along a single nanochannel. To also investigate how this average Δδ*V* obtained along a single channel varies *across several nominally identical channels* with the same depth, we have extracted the average Δδ*V* along four nominally identical nanochannels of each depth located all on the same chip and plot these values together with error bars that depict the span of average Δδ*V* (the smallest and largest obtained Δδ*V*) between four nominally identical nanochannels (Figure S10a (H_2_O_2_) and Figure S10b (NaCl)).

**Figure 5:**
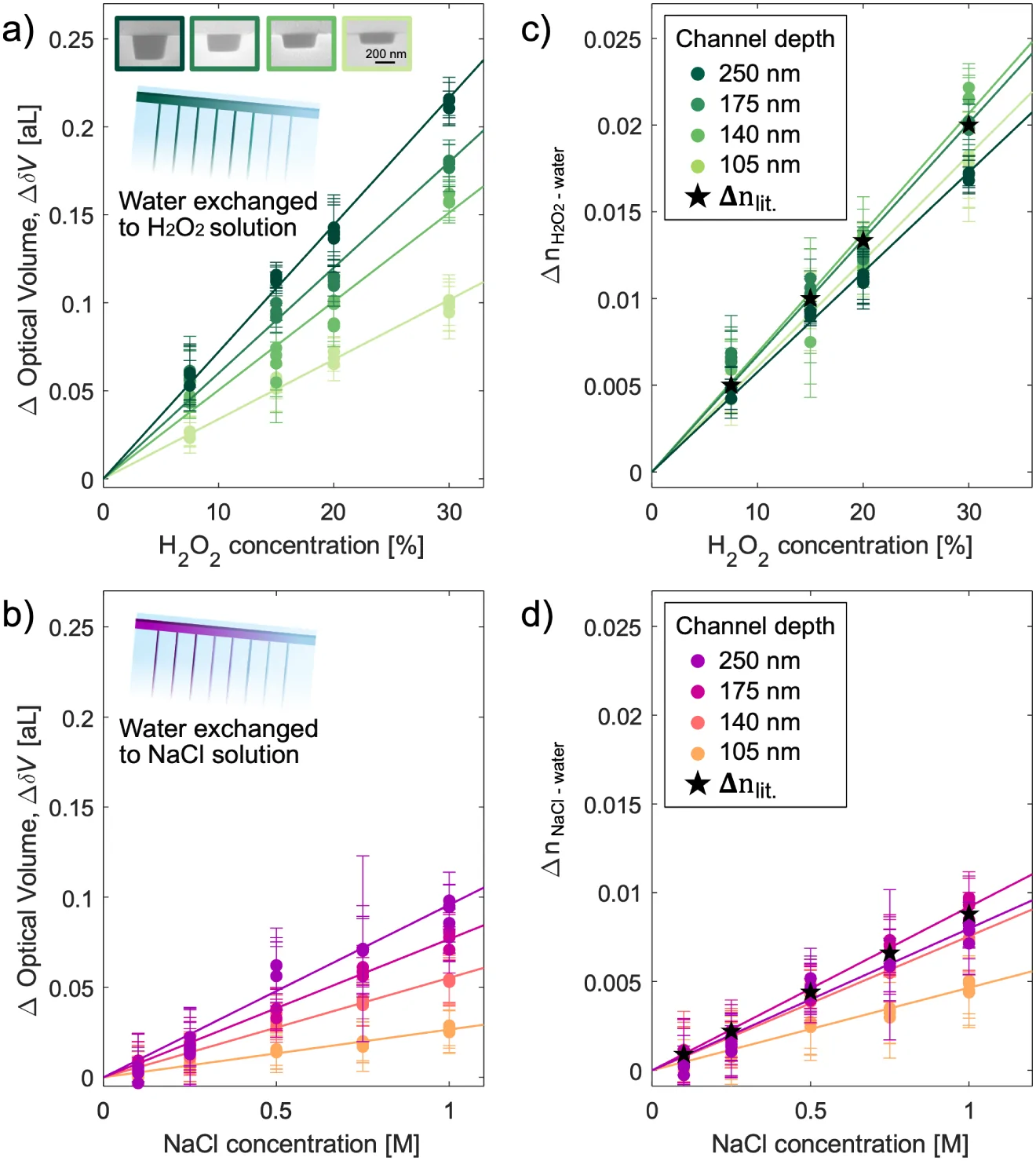
a) Δδ*V* averaged over the imaged 87 μm-long sections of four nanofluidic channels with different depths, i.e., 250 nm, 175 nm, 140 nm, and 105 nm, at a constant width of 350 nm, when exchanging their content from pure water to H_2_O_2_ solutions with concentrations indicated on the *x*-axis. The circular markers represent measurements of individual channels and are color-coded according to the channel depth. The error bars depict the standard deviation of Δδ*V* along the entire measured 87 μm-long nanochannel section. The inset depicts SEM cross-section images of the nanochannels with four different depths. b) Same as a) but for NaCl solutions with different concentrations depicted on the *x*-axis. c) $\Delta n_{H_{2}O_{2}-\mathrm{water}}$ as obtained by dividing the Δδ*V* values in a) with the physical volume of the corresponding nanochannel calculated using the mean cross-section area between the smallest and largest such area obtained from SEM image analysis of nominally identical nanochannels on the same chip (see SI section 1). The obtained values correspond well with Δ*n* values calculated based on the literature and the nominal concentrations of the used H_2_O_2_ solutions (black stars (★), see also SI Tables S2 and S3). The error bars in c) depict the standard deviation of $\Delta n_{H_{2}O_{2}-\mathrm{water}}$ extracted along the entire imaged 87 μm-long nanochannel section. d) Same as c) but for NaCl solutions with different concentrations depicted on the *x*-axis. Figure S10 depicts the same data but with error bars indicating the variation in average Δδ*V*, $\Delta n_{H_{2}O_{2}-\mathrm{water}}$ , and Δ*n*_NaCl‑water_, respectively, for four nominally identical nanochannels of each depth.

As a second aspect, from the average Δδ*V*–concentration relationship displayed in Figure 5a,b, it is apparent that Δδ*V* increases with larger nanochannel depths. By dividing Δδ*V* by the physical volume of the nanochannel calculated using the mean cross-section area between the smallest and largest values obtained from SEM image analysis of nominally identical nanochannels on the same chip and the length represented by one pixel (72.7 nm), the RI difference between two liquids, Δ*n_solution‑water_* = Δδ*V*_solution‑water_/*V*, can be calculated. Doing so for water and H_2_O_2_ solutions of various concentrations yields a linear “master curve” where the data points obtained from the four different nanochannel depths for the four different H_2_O_2_ concentrations (Figure 5c) line up reasonably well with the literature values summarized in SI Table 2. Similar analysis for the measurements with NaCl solutions (see Figure 5d) yields qualitatively very similar results in the sense that three out of four nanochannels line up well with the literature values. We note that in these plots, the error bars again depict the standard deviation of Δ*n*_solution‑water_ extracted along the entire imaged 87 μm-long nanochannel section. An additional perspective of the same Δ*n*_solution‑water_ data is shown in Figure S10c (H_2_O_2_) and Figure S10d (NaCl), where error bars instead depict the span of average Δ*n*_solution‑water_ (smallest to largest) obtained for 4 nominally identical nanochannels.

As the last aspect, it is interesting to put the found experimental errors/uncertainties into perspective. To this end, the standard deviation in Δ*n*_solution‑water_ along a nanochannel is on average 0.0017 RI units, whereas across 4 nominally identical nanochannels, it is on average 0.00076 RI units. This means that the main contribution to experimental uncertainty and therefore limit in resolution in RI (and consequently solute concentration) is the variation along a single nanochannel, most likely induced by local small variations in nanochannel cross-section dimensions due to, e.g., surface roughness or local imperfections in lid-bonding. The obtained RI standard deviation along a single nanochannel (0.0017 RI units) translates to a H_2_O_2_ concentration of 2.55% and a NaCl concentration of 0.16 M. While such errors could be considered significant in comparison to other optical methods used for measuring *relative RI changes* in nanofluidic channels,^31–33,37^ we argue that the advantage of employing CGM stems from the combination of performing *quantitative absolute RI measurements* (rather than relative changes), while maintaining a tiny sample volume offered by a single nanochannel.

As an example, to showcase the ability of CGM to monitor dynamic liquid composition—and thus RI—changes over time inside nanofluidic channels, we chose to investigate the diffusion of H_2_O_2_ into initially water-filled nanochannels. To prepare the experiment, we filled the microfluidic system on one side of the nanochannel with a 30% H_2_O_2_ aqueous solution and the one on the other side with pure Milli-Q water. Subsequently, we applied an overpressure of 800 mbar to the water and 100 mbar to the H_2_O_2_ side, respectively (Figure 6a). In this way, we establish a net convective flow of water through the nanochannels. Once the flow profile was established, we recorded a water baseline OPD image of the entire fluidic system. Subsequently, we increased the overpressure on the H_2_O_2_ reservoir to 400 mbar to also establish a flow of H_2_O_2_ through the corresponding microchannel but without pushing H_2_O_2_ into the nanochannels. Finally, we turned off all overpressure on both sides to establish a scenario without convective flow and thus only interdiffusion of the two liquids inside the nanochannel, which started immediately, and recorded a series of 10 frames over the course of 3.79 s. A corresponding OPD image obtained after 0.37 s of diffusion is depicted in Figure 6b and reveals both the expected different OPD values for the microfluidic channels filled with H_2_O_2_ (left) and water (right), respectively, and a clear OPD gradient along the nanofluidic channels from the left to the right, due to H_2_O_2_ diffusing into the water-filled nanochannels.

**Figure 6:**
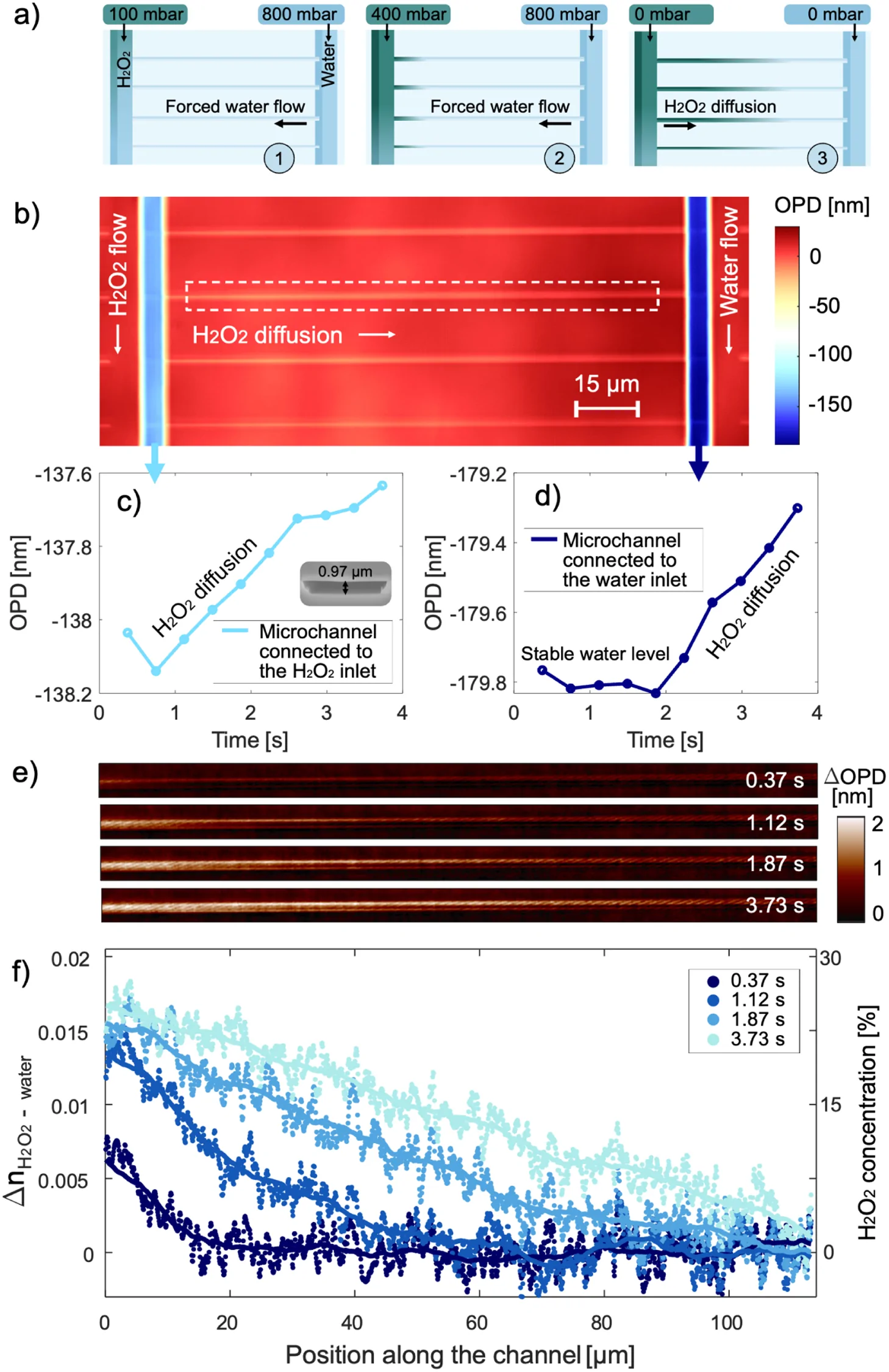
a) Schematic depiction of the experimental setup, sequence of applied overpressures, and correspondingly established flow and diffusion scenarios for the experiment of diffusing a 30% aqueous H_2_O_2_ solution into a water-filled nanofluidic channel. b) OPD image of the fluidic system depicted in a) obtained 0.37 s after the onset of H_2_O_2_ diffusion. The dashed rectangle depicts the nanochannel area that is analyzed in e) and f). c) OPD value averaged over the vertical H_2_O_2_-filled microchannel to the left in b) as a function of time from the start of the diffusion experiment . d) Same as c) for the water-filled microfluidic channel to the right in b). e) Time series of differential OPD images (Δδ*l* = δ*l* – δ*l*_water_) of a single nanochannel, i.e., images where we subtracted an OPD image of the water-filled nanochannel acquired prior to the diffusion experiment from all subsequent OPD images obtained during the diffusion experiment. f) Determined RI change, $\Delta n_{H_{2}O_{2}-water}$ , along the nanochannel at four different time stamps during the diffusion experiment (left *y*-axis). Evidently, a linear concentration gradient has formed along the nanochannel after 3.72 s extending from close to 0% H_2_O_2_ on the water side to close to 30% H_2_O_2_ on the H_2_O_2_ side (right *y*-axis). The solid lines serve as a guide to the eye. The individual data points correspond to individual pixels along the nanofluidic channel, which correspond to an area of 72.7 × 72.7 nm^2^ on the sample.

Focusing first on the H_2_O_2_-filled microchannel on the left, it can be noted that the average OPD over time (Figure 6c) immediately increases as the overpressures are turned off. The increase in OPD is reasonable since the water content in the left microchannel drops (and further increases the RI of the liquid in the channel) when the prior water flow from the nanochannels is terminated. Performing the same analysis on the microchannel filled with water (Figure 6d) reveals a delay of ∼2 s before the OPD starts to increase. This is because of H_2_O_2_ now having diffused all the way through the nanochannels and thereby entering the water-filled microchannel and changing its RI slightly later (Figure 6d). The RI change accumulated during the 10 frames (3.73 s) in both microchannels can be estimated from the observed change in average OPD, which in both cases is ∼0.5 nm, and the known microchannel depth of 0.97 μm using eq 1. This yields a RI change of Δ*n* = 0.0003 and translates into a H_2_O_2_ concentration increase of 0.6% in both microchannels.

As the next step, we analyze the H_2_O_2_ diffusion into the nanochannels and focus on the channel with 250 nm depth. To visualize the OPD change within the nanochannel, we constructed differential OPD images, i.e., images where we subtracted an OPD image of the water-filled nanochannel acquired prior to the diffusion experiment from all subsequent OPD images obtained during the diffusion experiment (Figure 6e). To subsequently quantify the RI change within the single nanochannel and track this change over time, we, frame by frame, calculate the OV for each pixel along the channel. The analyzed region of the OPD images is indicated by a white dashed rectangle in Figure 6b. From the extracted OV values, we calculate Δδ*V* = δ*V* – δ*V*_water_ along the channel using the measurement of the water-filled channel acquired prior to the diffusion measurements. Calculating Δδ*V* serves two purposes: (i) Δδ*V*_water_ serves as a baseline allowing us to compare the change with a known medium and (ii) the subtraction eliminates noise in the data stemming from impurities within the chip. From Δδ*V*, the RI change $\Delta n_{H_{2}O_{2}-\mathrm{water}}$ is determined by division with the physical volume section captured for each pixel along the channel (nanochannel cross-section × length represented by one pixel). Plotting the accordingly determined $\Delta n_{H_{2}O_{2}-\mathrm{water}}$ and H_2_O_2_ concentrations as a function of position along the nanochannel (the individual pixels of the camera along the nanochannel), for 4 different timestamps, i.e., after 0.37 s, 1.12 s, 1.87 s, and 3.73 s, reveals the establishment of a linear diffusion gradient along the nanochannel already after ∼2 s (Figure 6f). This is in very good agreement with the delay time for the onset of RI change determined above for the initially water-filled microchannel (c.f. Figure 6d). Furthermore, consulting the calculated absolute H_2_O_2_ concentrations along the gradient indeed reveals that it extends from close to 30% to close to 0%, which is reasonable since this matches the applied solutions to the inlet on each side of the chip.

As the last step, it is interesting to consider the smallest geometrical volume, i.e., the smallest section of a nanochannel, that we can resolve with our setup and from which we can determine a *local* concentration inside the nanochannel. Given the 480 nm spatial resolution of the microscope used, a diffraction-limited spot has a diameter corresponding to ∼7 pixels (area on the sample corresponding to a pixel = 72.7 × 72.7 nm^2^), which translates to a nanochannel section of 509 nm length. Combined with the cross-sectional area of the nanochannel used in this experiment, i.e., 350 × 250 nm^2^, this corresponds to a physical volume of ∼45 aL. Hence, replotting Figure 6f by averaging 7 adjacent pixels into one (spatially resolved) data point represents $\Delta n_{H_{2}O_{2}-\mathrm{water}}$ values for optically resolved nanochannel fractions that each correspond to a physical volume of ∼45 aL (SI Figure S11 and corresponding discussion). Focusing on the linear diffusion profile at 3.73 s, the minimum and maximum $\Delta n_{H_{2}O_{2}-\mathrm{water}}$ values for the first and last 45 aL section of the nanochannel are 0.0182 and −0.5 × 10^–4^, respectively. This corresponds to H_2_O_2_ concentrations of 27.3 and −0.734%. Accordingly, along a linear concentration gradient fitted to the experimentally measured concentration gradient, the difference in H_2_O_2_ concentration between two adjacent 45 aL nanochannel volume sections corresponds to 0.13% H_2_O_2_. Given the R^2^-value of 0.96 of the linear fit, this analysis is valid and enables the estimation of absolute solute concentration in nanochannel sections with 45 aL volume.

## Conclusions

In summary, we have demonstrated the use of CGM to quantitatively measure the RI of the liquid content of single nanofluidic channels with geometric cross-sections smaller than the diffraction limit of light. By integrating an OPD image over the area encompassing a nanochannel, we showed that the OV of an arbitrary section of a nanochannel can be obtained. Furthermore, we showed that the difference in OV between an area of the sample with and without a nanofluidic channel is proportional to the RI of the liquid inside the nanochannel and that this proportionality scales with nanochannel volume. As we demonstrated using the examples of transparent NaCl and H_2_O_2_ solutions, this proportionality can be used to determine the absolute concentrations and diffusion profiles of solutes inside a nanofluidic system. Specifically, we observed the formation of a linear RI gradient within 4 s along a nanofluidic channel with 250 × 350 nm^2^ nominal cross-sectional dimensions and 113 μm length which was connected to a pure-water-filled microfluidic channel on one side and a 30% H_2_O_2_-solution-filled microchannel on the other side. Using the developed mathematical formalism, this RI gradient could be converted into an absolute H_2_O_2_ concentration gradient with 509 nm spatial resolution along the nanochannel (this corresponds to 7 pixels in the used microscope setup which has a lateral spatial resolution of 480 nm). This means that H_2_O_2_ concentrations in adjacent nanochannel sections with a geometrical volume of only ∼45 aL could be measured. As a final point, we note that the accuracy of such measurements is strongly dependent on the quality of the acquired reference interferogram and a flat mounting of the sample. Therefore, we introduced an approach where OPD images of the same nanochannel, from sample interferograms recorded subsequently with the same reference interferogram, are compared to achieve precise measurements of the change in OV between each OPD image. In a wider perspective, this study demonstrates the applicability of CGM to the characterization of processes taking place in volumes smaller than the diffraction limit of light, such as, e.g., nanofluidic systems, nanopores, or nanocavities, as well as its ability to quantitatively resolve local absolute changes of RI and thus, e.g., solute concentrations inside single nanofluidic structures with volumes in the few-attoliter regime.

## Methods

### Fluidic Chip Fabrication

The fluidic chips were fabricated in the ISO Class 6 cleanroom facilities at Chalmers University of Technology, Gothenburg, Sweden, using 4 in., 1 mm-thick, double-side-polished (DSP), (100) silicon wafers (SiMat). Processing included (i) **wafer cleaning:** SC-1 (NH_4_OH:H_2_O_2_:H_2_O, 1:1:5, 80 °C, 10 min), 2% HF dip, and SC-2 (HCl:H_2_O_2_:H_2_O, 1:1:5, 80 °C, 10 min), with DI-water rinsing between steps; (ii) **thermal oxidation:** 2.15 μm wet thermal SiO_2_ at 1050 °C (Centrotherm furnace); (iii) **alignment-mark fabrication:** (iii.a) HMDS vapor priming (QS V 200 μ_B_, Vapor Prime), spin coating of a 2 μm S1813 positive photoresist (Shipley), hot plate bake (110 °C, 1 min), maskless laser lithography (MLA, Heidelberg Instruments; defocus 2, dose 200 mJ cm^–2^), and development in MICROPOSIT MF-319 (1 min); (iii.b) RIE (PlasmaTherm): O_2_ descum (15 s, 80 sccm, 50 W, 250 mTorr), CF_4_ SiO_2_ etch (50 min, 40 sccm, 100 W, 100 mTorr), and O_2_ plasma treatment to facilitate photoresist and fluorocarbon-residue removal (1 min, 80 sccm, 50 W, 250 mTorr); (iii.c) resist stripping in acetone (2 h), followed by IPA and DI-water rinsing and blow-drying; (iv) **nanochannel fabrication:** four EBL/RIE cycles for nominal channel depths of 200, 140, 110, and 80 nm, respectively, using PMMA masks with thicknesses three times the respective nominal etch depths; (iv.a) spin coating of 950k PMMA (MicroChem/Microlithography Chemicals Corp., MCC) and hot plate bake (180 °C, 5 min), EBL exposure (Raith EBPG 5200; 5 nA, 2 nm beam step size, 2600 μC cm^–2^, no proximity-effect correction), and development in IPA:H_2_O (93:7, 6 °C) under gentle agitation, with development time proportional to resist thickness (2 min for 200 nm channels); (iv.b) RIE (Oxford Plasmalab 100): O_2_ descum (5 s, 40 sccm, 40 W, 40 mTorr), SiO_2_ etch (30 sccm CF_4_, 20 sccm CHF_3_, 100 W, 20 mTorr) with etch time proportional to nominal channel depth (7 min 53 s for 200 nm channels), and O_2_ plasma treatment to facilitate PMMA and fluorocarbon-residue removal (15 s, 40 sccm, 40 W, 40 mTorr); (iv.c) PMMA stripping in acetone (2 h), followed by IPA and DI-water rinsing and blow-drying; (v) **microchannel fabrication:** photolithography, RIE, and resist stripping using the same process as for the alignment marks (iii); (vi) **inlet fabrication:** (vi.a) photolithography using a 8.5 μm AZ 4562 positive photoresist (Merck Performance Materials), hot plate bake (100 °C, 1 min), contact exposure (MA6, Süss MicroTec; hard-contact mode, 1 min), development in MF-322 (2 min), and hard bake (115 °C, 45 min); (vi.b) opening of the thermal SiO_2_ by RIE using the same process as in (iii.b), with a 70 min CF_4_ etch and without the final O_2_ plasma treatment; (vi.c) mounting for through-wafer DRIE on a 6 in. carrier wafer with 2 μm wet thermal SiO_2_, coated with 8.5 μm AZ 4562 and baked at 115 °C for 45 min, followed by mounting of the device wafer using vacuum oil; (vi.d) through-wafer inlet etching by high-rate Bosch DRIE (PlasmaPro 100, Oxford Instruments; 900 cycles); (vi.e) cleaning in piranha solution (130 °C, 10 min); (vii) **wafer bonding:** piranha cleaning (130 °C) of the processed silicon wafer and a 4 in. Borofloat 33 lid wafer (Siegert Wafer), O_2_-plasma surface activation (PlasmaTherm; 100 W, 250 mTorr), prebonding by bringing the activated surfaces into contact, and fusion bonding (Centrotherm furnace; 555 °C, 5 h, N_2_ atmosphere); and (viii) **dicing:** bonded wafers diced into 24 mm-wide octagonal fluidic chips using a 250-μm-wide resin-bonded diamond blade (Disco DAD3350; 25,000 rpm, 2 mm s^–1^ feed rate).

### Cross-Sectional SEM Imaging of Channels

After all optical experiments had been conducted, the fluidic chip was cut in half across the nanofluidic channels to enable SEM imaging (Zeiss Supra 60VP) of the nanochannel cross-sections. An acceleration voltage of 7 kV, a working distance of 6.6 mm, and a magnification of 100,000 X were used. A 5 Å gold layer was sputtered onto the sample prior to SEM imaging to increase the conductivity of the sample. The cross-sectional SEM images were analyzed with ImageJ to retrieve the cross-sectional area: 39000, 50000, 57000, and 82000 nm^2^. A more detailed description of the area analysis is presented in SI Section S1.

### Experiment Preparation

The finalized nanofabricated chip was submerged in an overnight piranha bath prior to use to remove potential contamination and blockages inside the fluidic system. A NaCl solution was prepared by mixing solid salt (Sigma-Aldrich, > 99.5% AR) with Milli-Q water and diluted into the desired concentrations. Similarly, H_2_O_2_ solutions were prepared by dilution of a 30% H_2_O_2_ solution in water (Carlo Erba). The injection of liquids into the fluidic chip mounted in the chip holder was done with syringes with blunt needles from Braun.

### Microscopy and Illumination

Wavefront images were acquired using a custom-built inverted reflection microscope. A red LED at a central wavelength of 620 nm (SOLIS-620D, Thorlabs) was focused onto the back focal plane of the objective (40x, 1.35 NA, UApoN340, Olympus) to produce a quasi-plane-wave illumination at the sample plane. Wavefront images were acquired using quadriwave lateral shearing interferometry (QLSI), consisting of the association of a regular camera with a 2D diffraction grating (aka cross-grating), separated by a millimeter distance. We used a commercial QLSI system including a Zyla 5.2 camera (Andor) and a QLSI grating with a pitch of 19.5 μm, separated from the camera by a telecentric relay lens of magnification 2x (Sid4Element, Phasics). The grating-to-sensor distance was calibrated at λ = 620 nm to be 0.50 mm.

### Data Analysis

OPD images are constructed by treating sample interferograms (nanofluidic system) and reference interferograms (of the SiO_2_ substrate situated next to the nanofluidic system) with the PhaseLab toolbox we developed in MATLAB.^58^ In addition, all images were treated with a background correction algorithm based on Legendre polynomials that is part of the PhaseLab library. From the OPD images, a region of interest (ROI) spanning ∼5.5 × 87.2 μm^2^ (corresponding to 75 × 1200 pixels) is selected such that a nanochannel is centered and the edges are far (∼10 μm) from the microchannel on each side. Next, a linear background subtraction is performed row by row of pixels, using an average OPD value of the background. Half of the pixels used for representing the background are situated within the ROI containing the nanochannel, while the rest are right outside the border of the said ROI. This entails the construction of a linear function based on the average OPD value (of 14 pixels, 7 of which are within the ROI and 7 outside) on each side of the profile (30 pixels away from the center pixel), that then is subtracted from the middle region of the profile. To calculate the optical volume (OV) for each row, we integrate the image with regards to the width of each row, by summing the OPD of the pixels and multiplying with the area that each pixel represents (72.7 × 72.7 nm^2^). From this 1D array of data, the OV variations along a nanochannel can be reconstructed (as in, e.g., Figure 3f) or can be used to represent the entire channel by averaging it to a length representing one pixel (as displayed in Figure 4c). To calculate Δδ*V*, as presented in Figure 5ab, we have used two OPD images obtained with the same reference interferogram.

## Supplementary Material



## Data Availability

The underlying data for this publication is available at Zenodo, DOI: 10.5281/zenodo.20846844.

## Notes

Nanochannel cross-sections, refractive index estimation, optical volume, reproducibility between 4 nominally identical channels of each depth, and resolving H_2_O_2_ concentration in diffraction-limited sections of the nanochannel with a 45 aL physical volume (PDF)
