# Supplementary material for: Monitoring Liquid Concentration Changes in Nanofluidic Channels Using Cross-Grating Wavefront Microscopy
Source: ACS Nano. 2026 Oct 15;20(42):28565–75. doi: 10.1021/acsnano.6c12559 (PMC13718605; doi:10.1021/acsnano.6c12559)
Supplement: Supplementary file 1 [file nn6c12559_si_001.pdf]

Supplementary Information:

Monitoring Liquid Concentration Changes in Nanofluidic Channels using Cross-Grating Wavefront Microscopy

Lova Wilske[1], Baptiste Marthy[2], Puvaneswari Teluchina-Appadu[1], Joachim Fritzsche[1], Guillaume Baffou[2,] and Christoph Langhammer[1,*]*

[1]Department of Physics and Astronomy, Chalmers University of Technology; SE-412 96 Gothenburg, Sweden

[2]Institut Fresnel, CNRS, Aix-Marseille University, Centrale Med, Marseille, France

*Corresponding authors: guillaume.baffou@fresnel.fr; clangham@chalmers.se

1. Nanochannel cross-section determination from SEM images

To estimate the geometrical cross-sections of nanochannels, the fluidic chip was diced in half, after all experiments had been conducted, and its cross-section imaged with SEM. Prior to imaging, the fluidic chip cross-section was sputtered with a 0.5 nm thick Au layer to reduce sample charging from the electron beam during imaging. The SEM images were acquired with an acceleration voltage of 7 kV, a working distance of 6.6 mm, and a magnification of 100 000 x. By analyzing the SEM images with ImageJ, we retrieved two different areas – a minimum and a maximum cross-section area. This approach was used to capture the uncertainty in the SEM images cause by minor charging effects, image blurring and contrast issues, and to showcase the variety in cross-section area between nominally identical nanochannels. The results from the analyzed images are presented in **Table S1** and the images are displayed in **Figure S1-S4**. The average value between the smallest minimum area and the largest maximum area found for each nomina nanochannel depth from multiple images, was employed in the calculations of refractive index for the plots in **Figure 5** and **6** in the main text.

Table S1. *Nanochannel cross-section areas extracted from SEM images. Each given value represents the smallest and largest areas retrieved from the analysis performed on 2-4 cross-section SEM images of 2- 4 nominally identical nanochannels.*

	**depth 1**	**depth 2**	**depth 3**	**depth 4**
**Min area [nm^2]**	34000	45000	50000	76000
**Max area [nm^2]**	44000	55000	64000	88000
**Average between min and max area [nm^2]**	39000	50000	57000	82000
**# channels included in analysis**	4	2	2	3

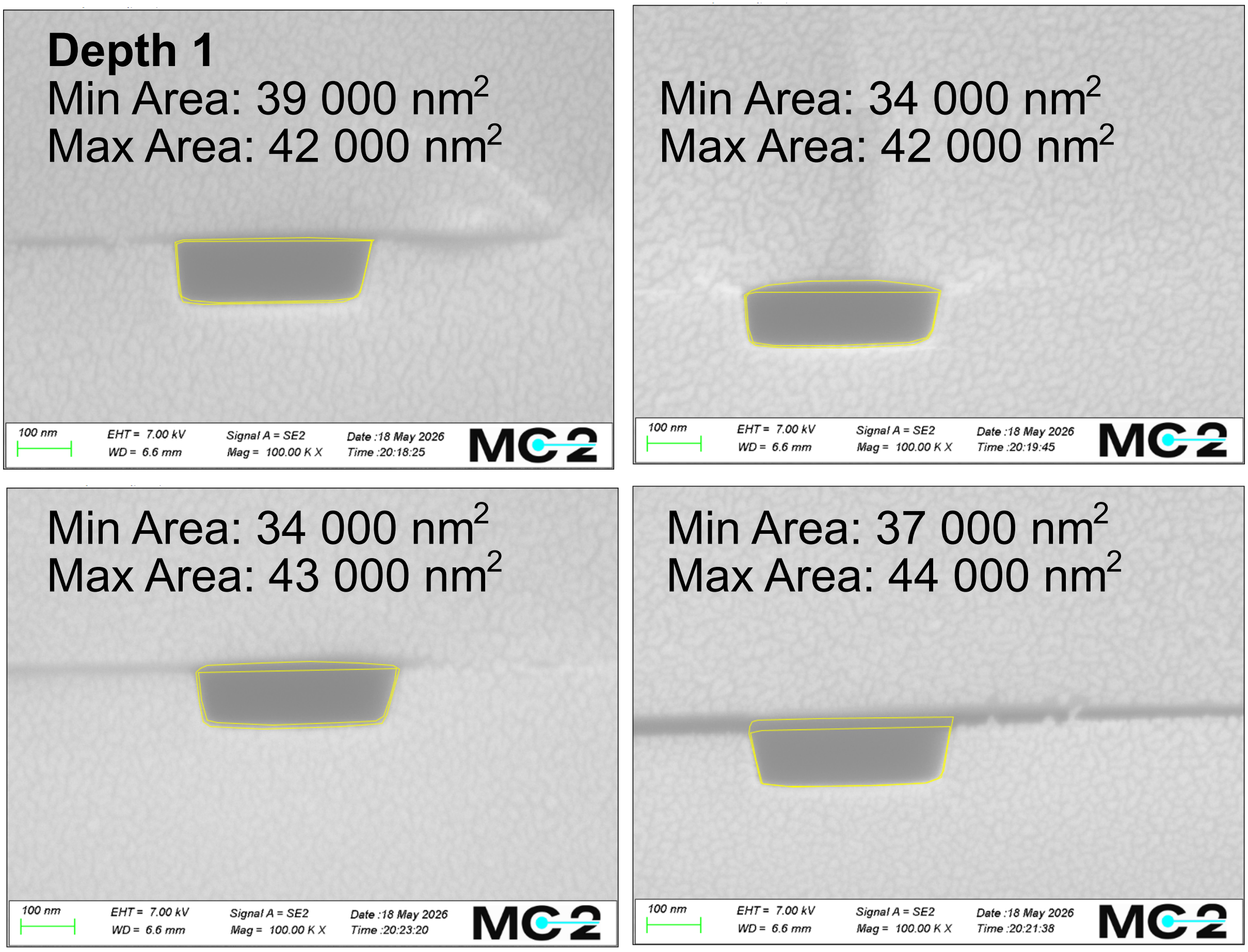

Figure S1. *Four cross-sectional SEM images of nanochannels with the same nominal depth (Depth 1: ~105 nm). The marked areas display the regions from which the minimum and maximum area of each image were retrieved.*

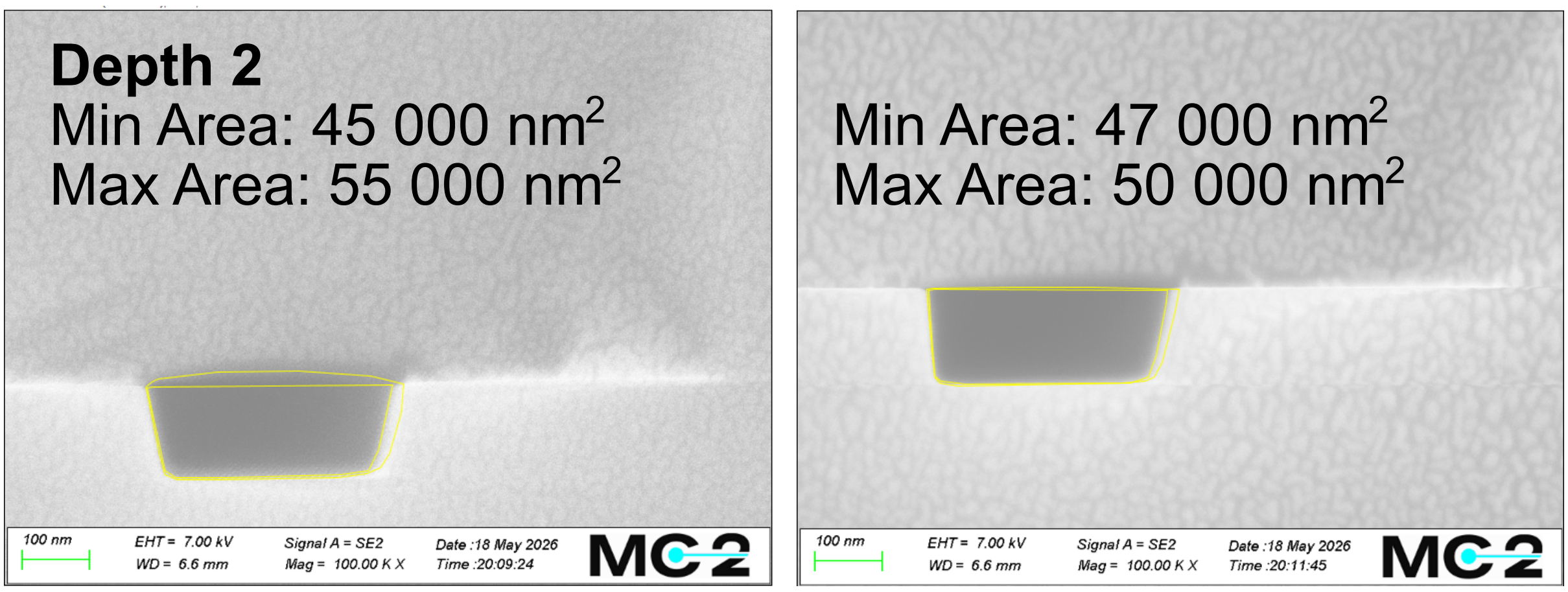

Figure S2. *Two cross-sectional SEM images of nanochannels with the same nominal depth (Depth 2: ~140 nm). The marked areas display the regions from which the minimum and maximum area of each image were retrieved.*

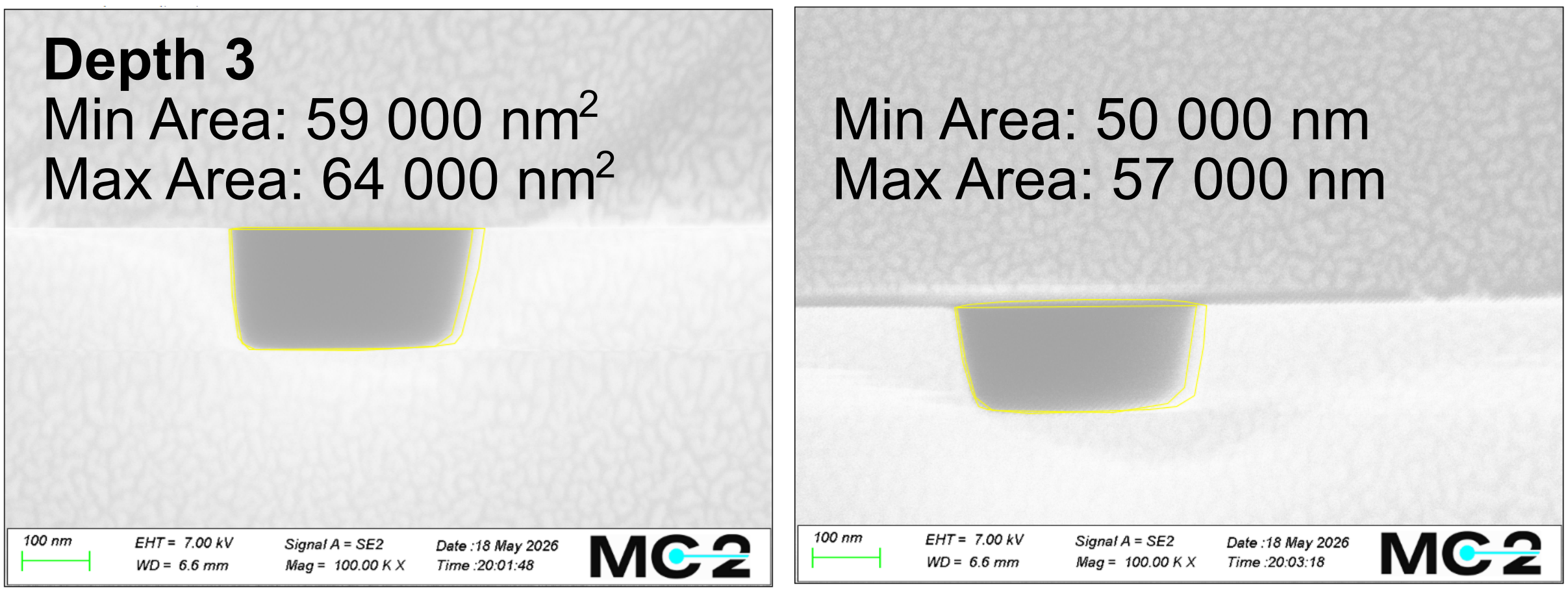

Figure S3. *Two cross-sectional SEM images of nanochannels with the same nominal depth (Depth 3: ~175 nm). The marked areas display the regions from which the minimum and maximum area of each image were retrieved.*

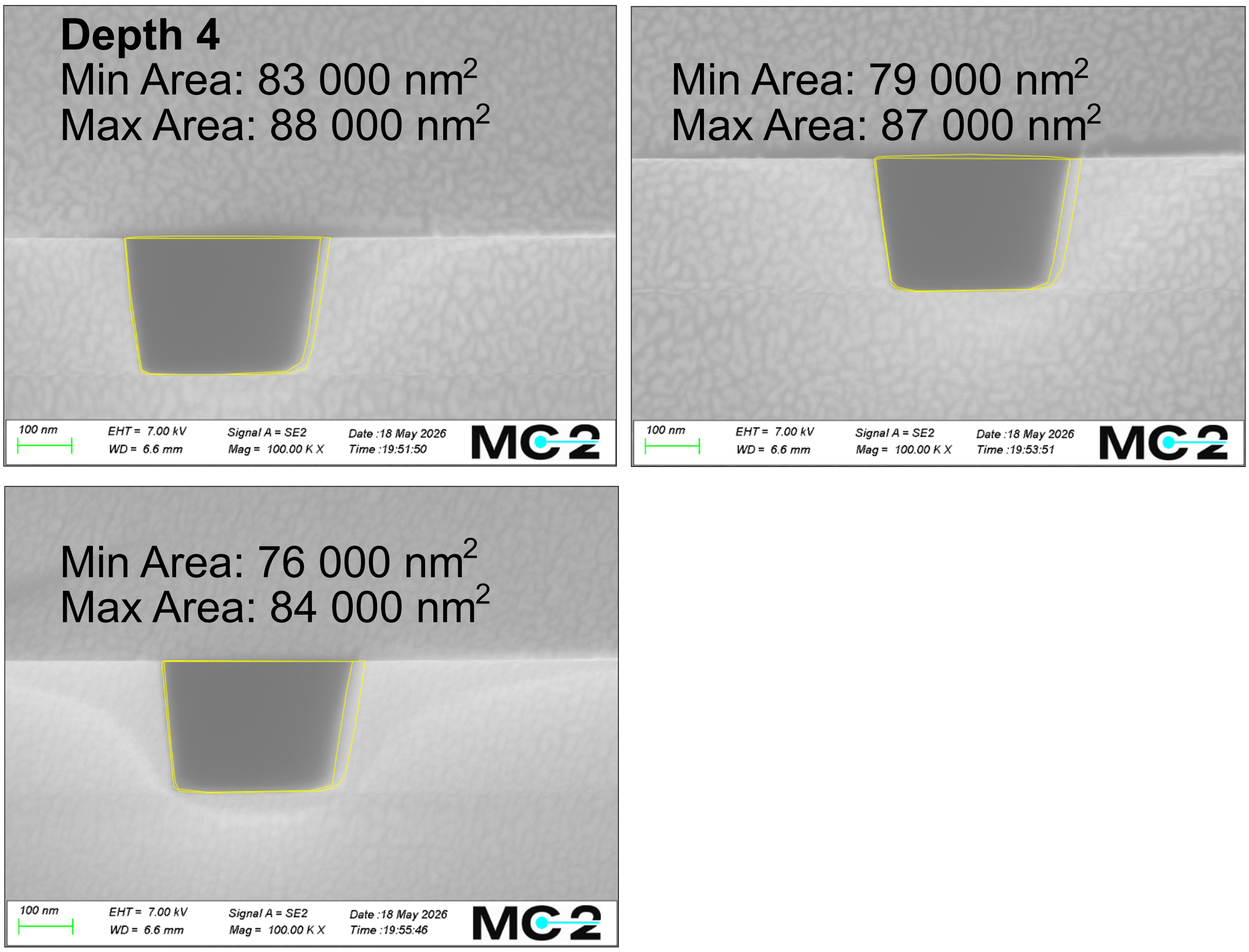

Figure S4. *Three cross-sectional SEM images of nanochannels with the same nominal depth (Depth 4: ~250 nm). The marked areas display the regions from which the minimum and maximum area of each image were retrieved.*

2. Refractive index estimation

A linear dependence between refractive index and concentration is assumed. The refractive index of a liquid C, comprised of a mixture of liquid A and B can thus be calculated by interpolation

$$n_C = n_A + \frac{c_C - c_A}{c_B - c_A}(n_B - n_A), \quad (1)$$

Where c designates the concentration of a species. Through interpolation, the refractive index, n, and the refractive index difference compared to water, Δn, of H_2O_2 in aqueous solution and NaCl aqueous solution at various concentrations were obtained, and are presented in **Table S2**, and **Table S3**, respectively. The tabulated values correspond to the experimental conditions of room temperature and 620 nm illumination wavelength.

Table S2. *Refractive index for H_2O_2 aqueous solutions at different concentrations.*

c [%]	n	Δn
30	1,352*	0,020
20	1,345	0,013
15	1,342	0,010
7,5	1,337	0,005
0	1,332**	0,000

Tabel S3. *Refractive index for NaCl aqueous solutions at different concentrations.*

NaCl concentration [mM]	n	Δn
4280	1,3780***	0,0460
3000	1,3578	0,0278
2000	1,3486	0,0185
1000	1,3393	0,0093
750	1,3370	0,0070
500	1,3346	0,0046
250	1,3323	0,0023
100	1,3309	0,0009
0	1,332**	0

*n for 30 % H_2O_2 at 27 C, at 589 nm is 1.3578, and at 644 nm is 1.3516.[1]

**n for pure water at 25 C and 600 nm is 1.332.[2]

***n for 25 % (4280 mM) NaCl at 589 nm is 1,3680.[3]

3. Optical volume

The optical volume (OV) is calculated by integrating optical path distance (OPD) over an area of an OPD image. To reduce noise from the data when extracting the OV of a nanofluidic channel, the OPD background was subtracted from a region of interest spanning 5.5 ×87.6 μm^2 and containing a nanochannel at the center. OV measurements of 16 nanochannels with four different depths filled with water and 3 M NaCl solution are shown in **Figure S5**.

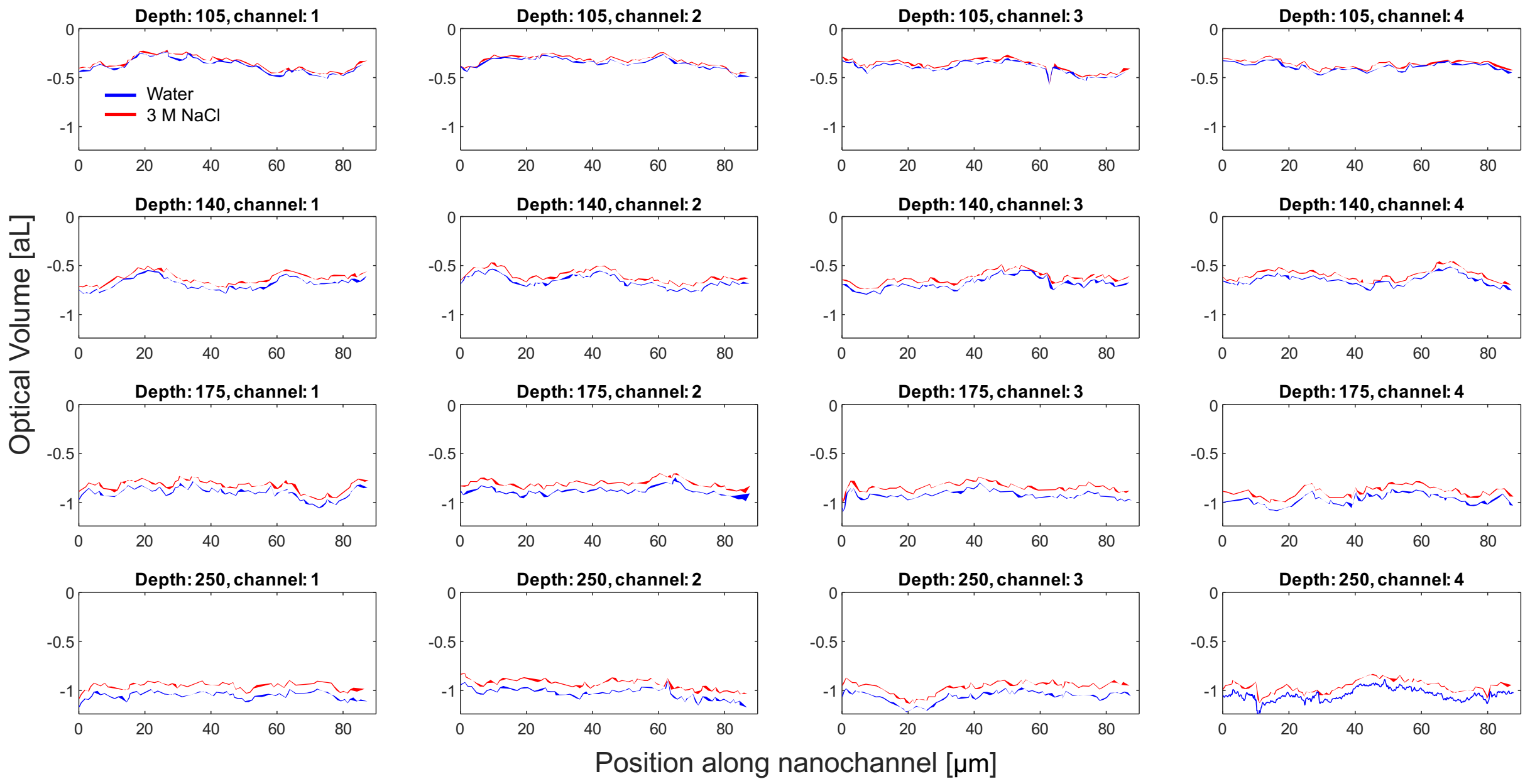

Figure S5. *δV measurements along an 87 μm stretch of nanochannel. The blue lines correspond to water filled channels and the red lines to channels filled with 3 M NaCl solution. The width of all channels is 350 nm, and the depth is given in the title of each graph.*

The average OPD cross-cut profiles of background subtracted nanochannels are shown in **Figure S6** and **S7**, for measurements of nanochannels filled with water-H_2O_2 solutions and water-NaCl solution respectively. The OV shifts along the nanochannels generated by exchanging water to a H_2O_2 or NaCl solution are shown in **Figure S8** and **S9**.

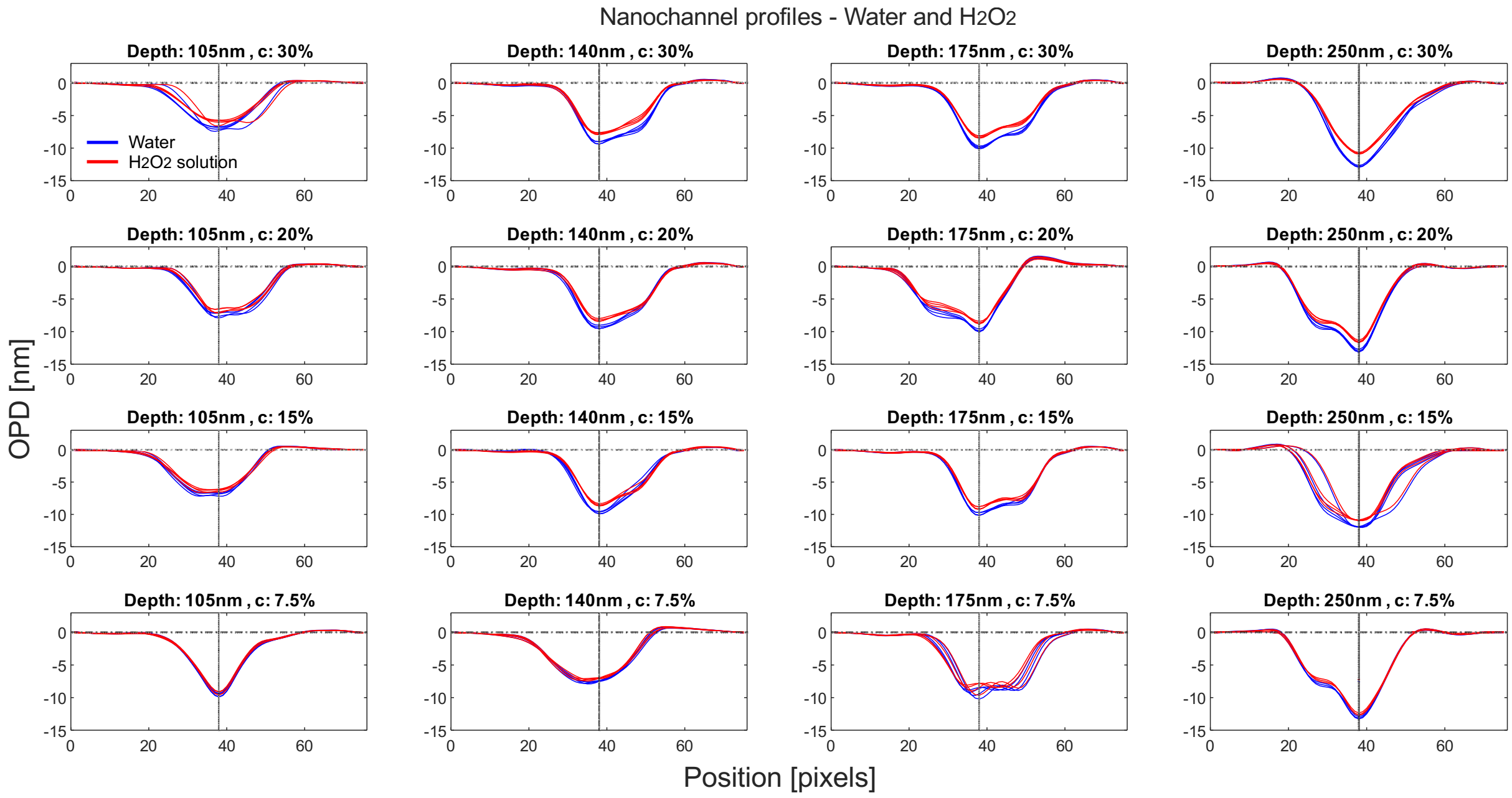

Figure S6. *Average OPD profile across nanofluidic channels. The blue lines in each graph display the profiles of four water filled channels and red of four channels filled with H_2O_2 solution. The title above each graph indicates the nanochannel depth and the concentration in %, c, of the H_2O_2 solution. The graph in the lower right corner only includes measurement of 3 nanochannels.*

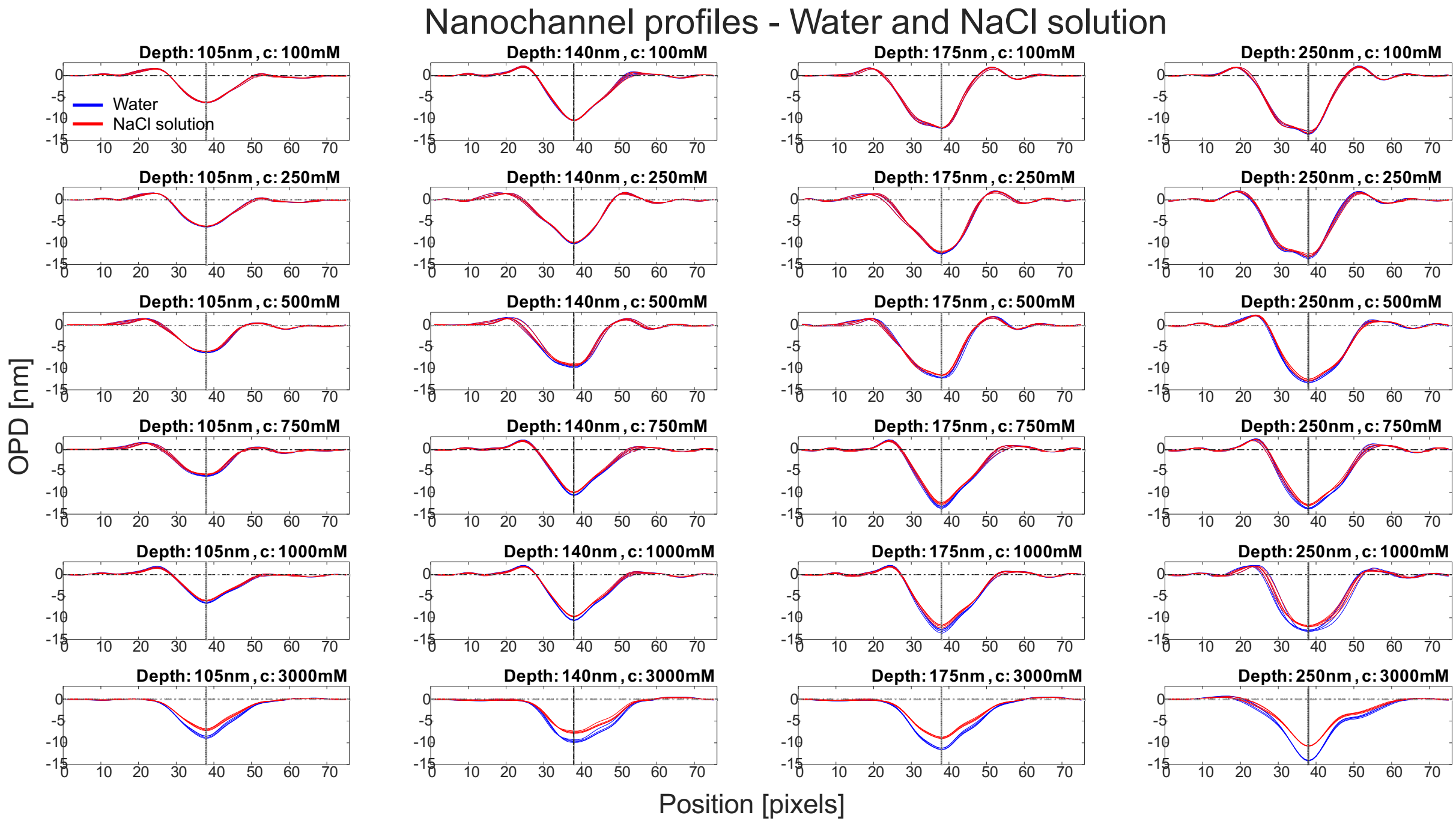

Figure S7. *Average OPD profile across nanofluidic channels. The blue lines in each graph display the profiles of four water filled channels and red of four channels filled with NaCl solution. The title above each graph indicates the nanochannel depth and the concentration, c, of the NaCl solution.*

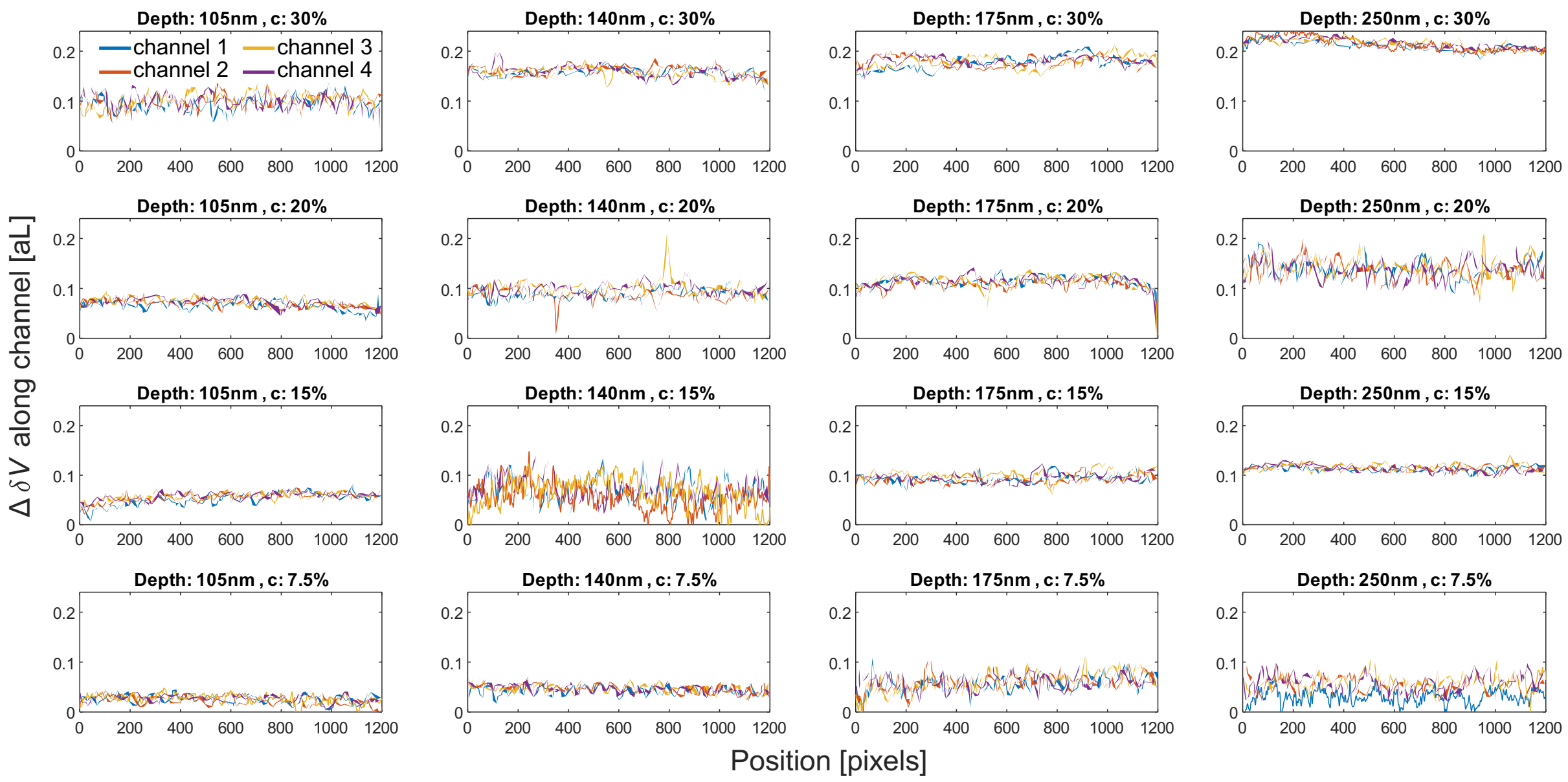

Figure S8. *ΔδV measurements along an 87 µm stretch of nanochannel (1200 pixels). ΔδV is generated by exchanging the channel medium from water to a* H_2O_2 *solution. The depth of each channel and the* H_2O_2 *concentration, c, is indicated in the title of each graph.*

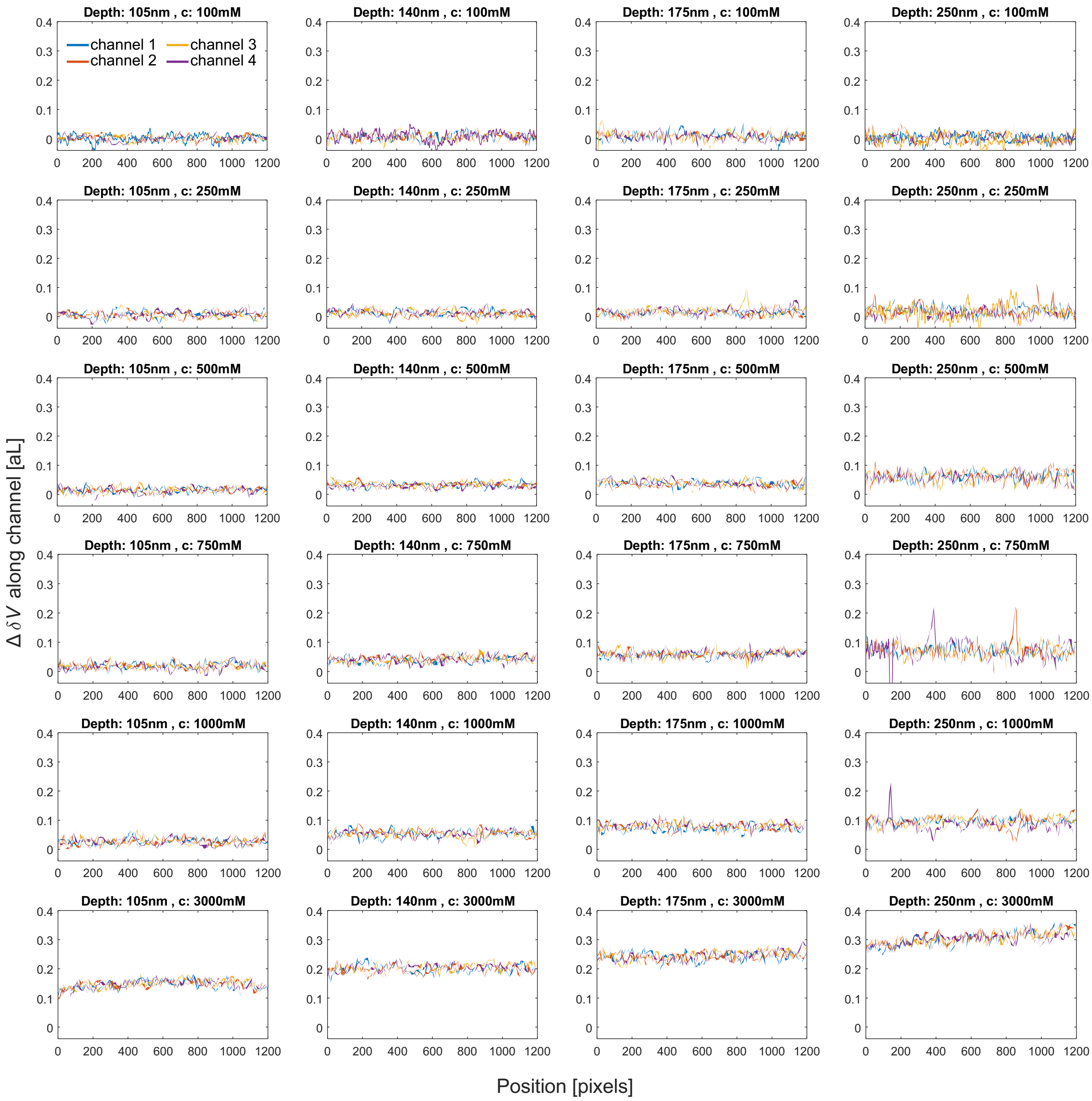

Figure S9. *ΔδV measurements along an 87 µm stretch of nanochannel (1200 pixels). ΔδV is generated by exchanging the channel medium from water to a NaCl solution. The depth of each channel and the NaCl concentration, c, is indicated in the title of each graph.*

4. Reproducibility between 4 nominally identical channels of each depth

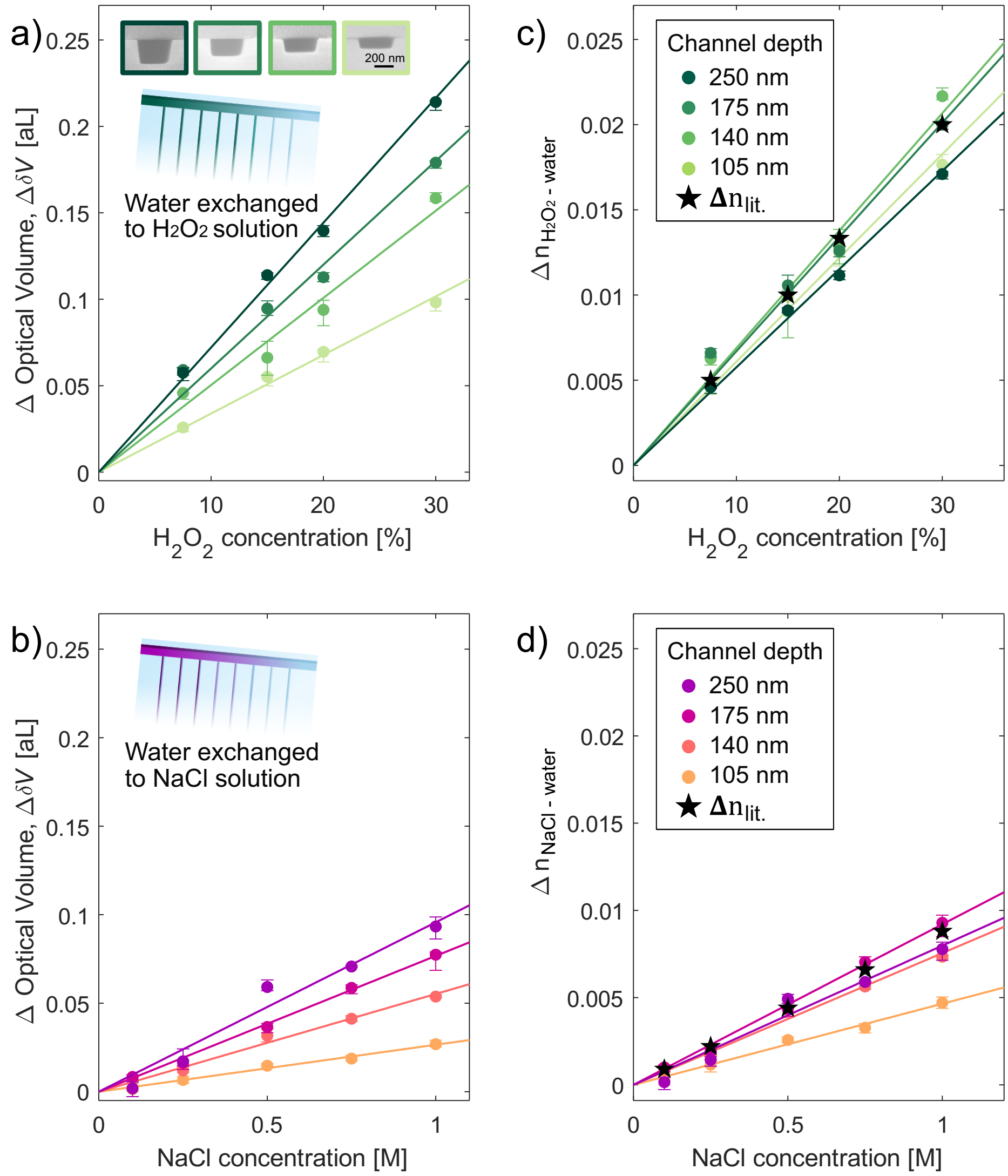

Figure S10. *a) ΔδV averaged over the imaged 87 µm long sections of nanofluidic channels with different depths, i.e., 250 nm, 175 nm, 140 nm and 105 nm, at constant width of 350 nm, when exchanging their content from pure water to H_2O_2 solutions with concentrations indicated on the x-axis. The circular markers represent the average ΔδV response from a set of 4 different channels (averaged for each channel along the 87 µm long imaged section) with the same nominal dimensions for each depth. The error bars depict the span of average ΔδV (minimum to maximum average ΔδV) for these 4 channels. The inset depicts SEM cross-section images of the nanochannels with four different depths. b) Same as a) but for NaCl solutions with different concentrations depicted on the x-axis. c) $\Delta n_{H_2O_2-water}$ as obtained by dividing the ΔδV values in a) with the physical volume of the corresponding nanochannel calculated using the mean cross-section area between the smallest and largest such area obtained from SEM image analysis of nominally identical nanochannels on the same chip (see SI section 1). The obtained values correspond well with Δn values calculated based on the literature and the nominal concentrations of the used H_2O_2 solutions (black stars (⋆), see also* ***SI table 2, 3****). The error bars depict the span (minimum to maximum) of $\Delta n_{H_2O_2-water}$ measured for the 4 channels of nominally identical dimensions for each depth. d) Same as c) but for NaCl solutions with different concentrations depicted on the x-axis.*

5. Resolving H_2O_2 concentration in diffraction limited sections of the nanochannel with 45 aL physical volume

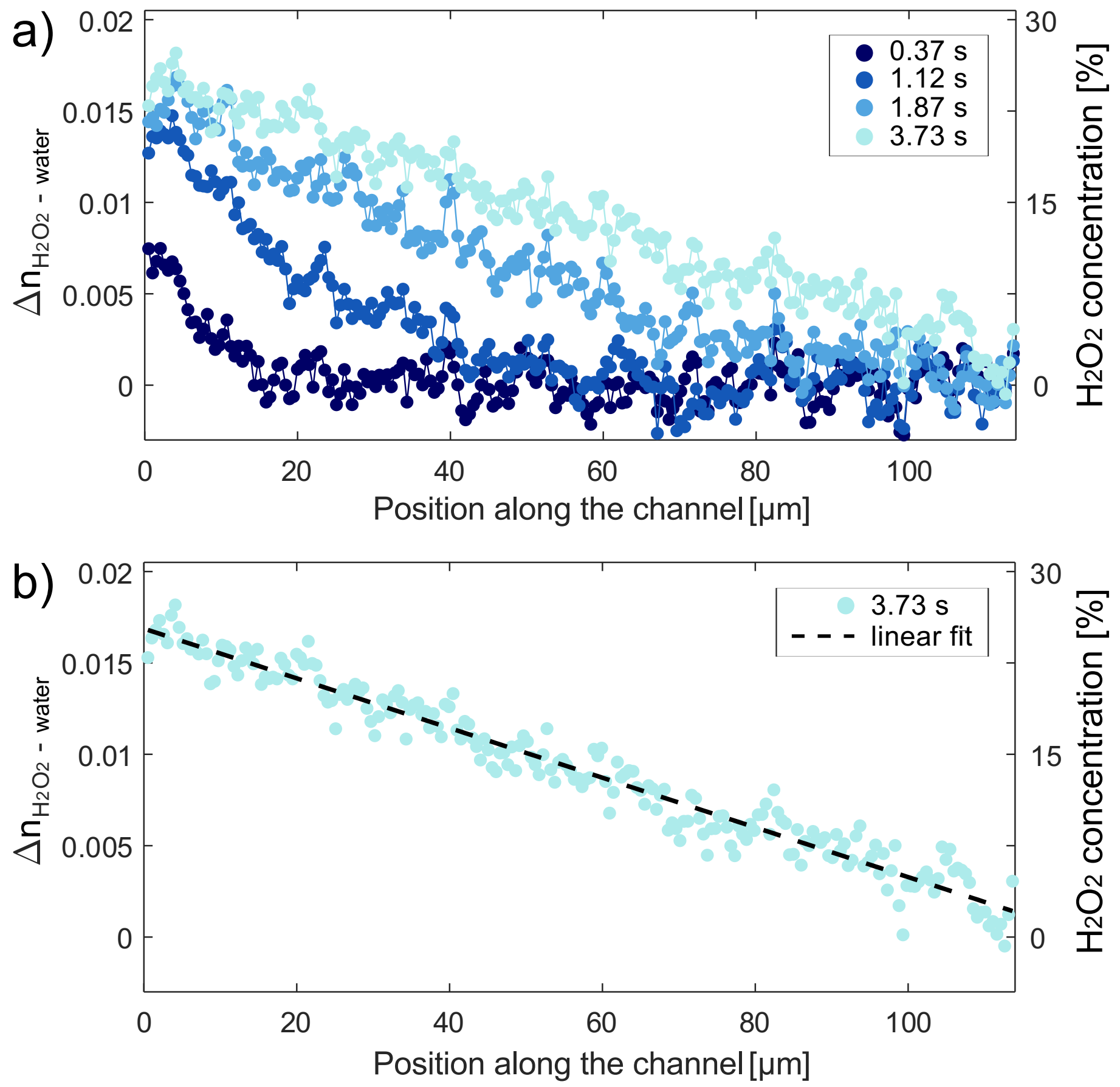

Figure S11. *a) $\Delta n_{\mathrm{H_2O_2-water}}$ measured along a 113 µm long nanofluidic channel of width 350 nm and depth 250 nm. Initially, the channel is filled with water, but as time progresses, a 30 % H_2O_2 aqueous solution is diffusing into the channel. Each data point represents the average from 7 adjacent pixels, which corresponds to a geometrical area along the nanochannel larger that the lateral resolution of the microscope (480 nm). Hence, each point represents $\Delta n_{\mathrm{H_2O_2-water}}$ from the smallest spatially resolved fraction of the nanochannel which has a geometrical volume of 45 aL. b) Linear fit to the experimentally measured concentration gradient at $t = 3.73$ s with $R^2 = 0.96$.*

References

1. Tonkyn, R. G. No Title. https://webbook.nist.gov/chemistry/silmarils-liquids-n-k/docs/META.MIR.MIX.H2O2-30pct-in-H2O.LIQ.SIGMA-ALDRICH.PNNL316541c.pdf (2017).

2. Hale, G. M. & Querry, M. R. Optical Constants of Water in the 200-nm to 200-µm Wavelength Region. *Appl. Opt.* **12**, 555–563 (1973).

3. Tan, C.-Y. & Huang, Y.-X. Dependence of Refractive Index on Concentration and Temperature in Electrolyte Solution, Polar Solution, Nonpolar Solution, and Protein Solution. *J. Chem. Eng. Data* **60**, 2827–2833 (2015).
